# CpG traffic lights are markers of regulatory regions in human genome

**DOI:** 10.1186/s12864-018-5387-1

**Published:** 2019-02-01

**Authors:** Anna V. Lioznova, Abdullah M. Khamis, Artem V. Artemov, Elizaveta Besedina, Vasily Ramensky, Vladimir B. Bajic, Ivan V. Kulakovskiy, Yulia A. Medvedeva

**Affiliations:** 1 Institute of Bioengineering, Research Center of Biotechnology, Russian Academy of Sciences, Moscow, 119071 Russia; 2 Computational Bioscience Research Center (CBRC), Computer, Electrical and Mathematical Sciences and Engineering (CEMSE) Division, King Abdullah University of Science and Technology (KAUST), Thuwal, 23955-6900 Saudi Arabia; 3 Faculty of Bioengineering and Bioinformatics, Lomonosov Moscow State University, Moscow, 119991 Russia; 4 Institute for Information Transmission Problems (Kharkevich Institute), Russian Academy of Sciences, Moscow, 127051 Russia; 5 Moscow Institute of Physics and Technology, Dolgoprudny, Moscow Region, 141701 Russia; 6 Engelhardt Institute of Molecular Biology, Russian Academy of Sciences, Moscow, 119991 Russia; 7 Institute of Mathematical Problems of Biology RAS - the Branch of Keldysh Institute of Applied Mathematics of Russian Academy of Sciences, Pushchino, 142290 Moscow Region Russia; 8 Vavilov Institute of General Genetics, Russian Academy of Sciences, Moscow, 119991 Russia

**Keywords:** CpG traffic lights, DNA methylation, Transcription regulation, Enhancers, CAGE, Chromatin states, NRF1, ETS, STAT, IRF

## Abstract

**Background:**

DNA methylation is involved in the regulation of gene expression. Although bisulfite-sequencing based methods profile DNA methylation at a single CpG resolution, methylation levels are usually averaged over genomic regions in the downstream bioinformatic analysis.

**Results:**

We demonstrate that on the genome level a single CpG methylation can serve as a more accurate predictor of gene expression than an average promoter / gene body methylation. We define CpG traffic lights (CpG TL) as CpG dinucleotides with a significant correlation between methylation and expression of a gene nearby. CpG TL are enriched in all regulatory regions. Among all promoters, CpG TL are especially enriched in poised ones, suggesting involvement of DNA methylation in their regulation. Yet, binding of only a handful of transcription factors, such as NRF1, ETS, STAT and IRF-family members, could be regulated by direct methylation of transcription factor binding sites (TFBS) or its close proximity. For the majority of TF, an alternative scenario is more likely: methylation and inactivation of the whole regulatory element indirectly represses functional TF binding with a CpG TL being a reliable marker of such inactivation.

**Conclusions:**

CpG TL provide a promising insight into mechanisms of enhancer activity and gene regulation linking methylation of single CpG to gene expression. CpG TL methylation can be used as reliable markers of enhancer activity and gene expression in applications, e.g. in clinic where measuring DNA methylation is easier compared to directly measuring gene expression due to more stable nature of DNA.

**Electronic supplementary material:**

The online version of this article (10.1186/s12864-018-5387-1) contains supplementary material, which is available to authorized users.

## Background

Epigenetic regulation of gene expression has been thoroughly investigated over last decades. DNA methylation, usually in CpG context, is probably the most well-studied mechanism of epigenetic regulation. DNA methylation is linked to many normal and pathological biological processes: organism development, cell differentiation, cell identity and pluripotency maintenance (reviewed in [1–3]), aging [4], memory formation [5, 6], responses to environmental exposures, stress and diet [7–9]. Abnormalities in DNA methylation play an important role in various diseases, including metabolic [10], cardiovascular [11], neurodegenerative [12, 13] diseases and cancers (reviewed in [14]). For about a decade, DNA demethylating drugs (Decitabine, Azacytidine) are used in clinic for the treatment of acute myeloid leukemia and myelodysplastic syndrome [15]. Recent advances in site-specific editing of DNA methylation [16] suggest DNA methylation as a promising target for non-invasive therapies against diseases linked to aberrant methylation.

Functionally, DNA methylation of promoter regions is tightly associated with repression of transcription initiation, while high levels of gene body methylation, on the contrary, are linked to the increased gene expression (reviewed in [17]). Enhancers, distant regulatory regions, that contribute to the establishment of the correct temporal and cell-type-specific gene expression pattern, have been shown to initiate transcription of short RNAs [18]. Therefore, it is no surprise that DNA methylation also regulates the enhancer functioning [19–22].

Methods based on bisulfite sequencing allow detection of single cytosine methylation. Yet, in downstream bioinformatic analysis, methylation levels of several dozens of cytosines are often averaged to increase statistical power [23, 24]. At the same time, multiple examples show that changes in methylation of a single CpG can affect transcription [25–39]. Recently, we have shown that methylation of particular single CpG dinucleotides are tightly linked to gene expression [40]. We have called such positions CpG traffic lights (CpG TL) and have demonstrated a strong negative selection against them in computationally predicted transcription factor binding sites. In the current study we show enrichment of CpG TL in regulatory elements of different types: in transcription start sites (TSS), in particular, in poised promoters, as well as in enhancers and regions with active chromatin marks. Although CpG TL may regulate transcription factors, co-factors and epigenetic regulators, binding of only a handful of transcription factors could be regulated by direct methylation of a CpG TL within a transcription factor binding site (TFBS). For the majority of TF, an alternative scenario is more likely: inactivation of the whole regulatory element via DNA methylation repress TF binding indirectly; and CpG TL are reliable markers of inactivation. We believe that CpG TL provide a promising insight into mechanisms of enhancer activity and gene regulation linking methylation of single CpG to gene expression.

## Results

### CpG traffic lights detection

**Fig. 1:**
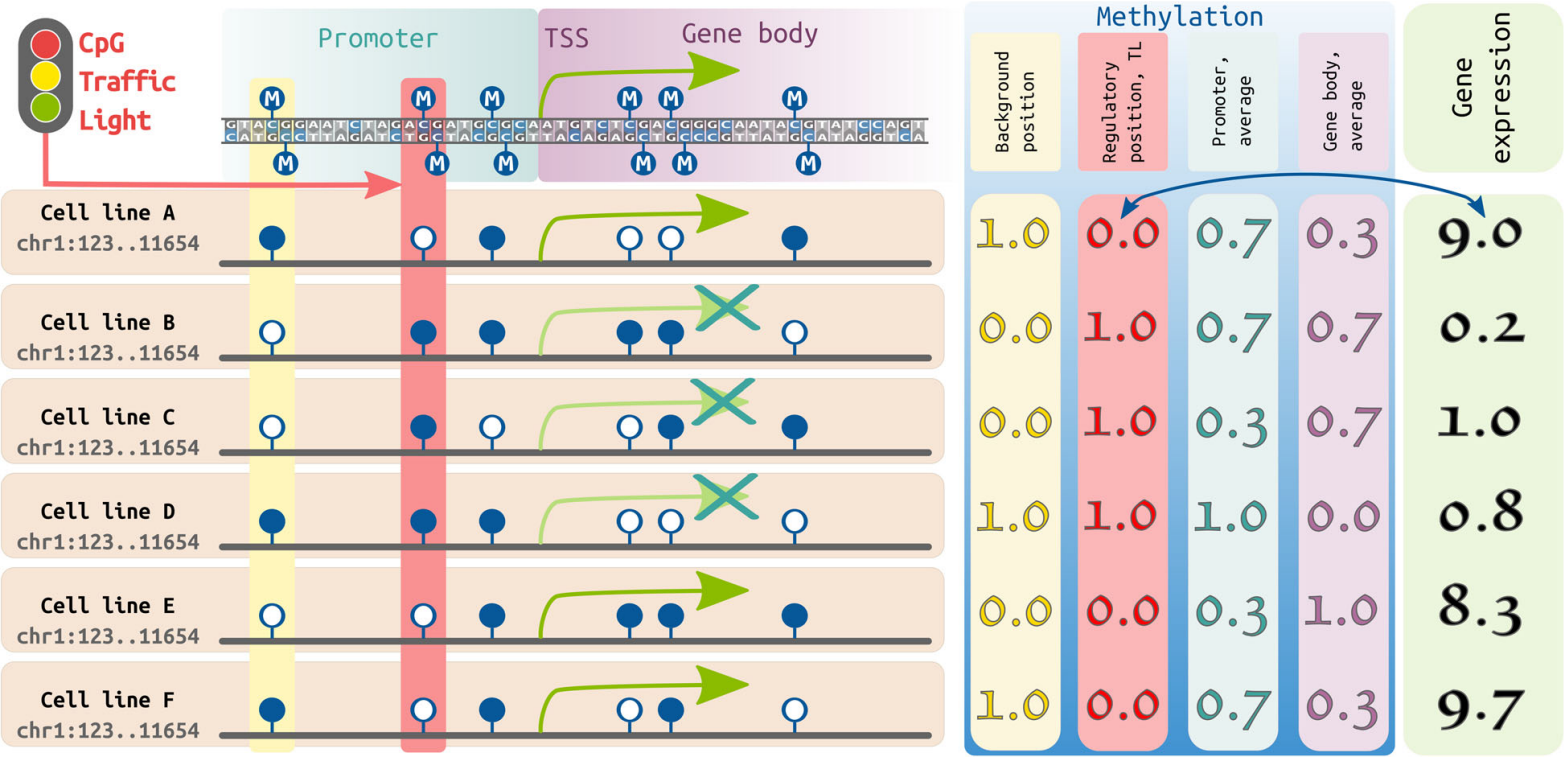
Schematic representation of a CpG traffic light detection. **Left panel**. Suppose we analyze a particular genomic region (chr1:123..11654), which contains for simplicity one gene. For each CpG in this region and the gene we have methylation and expression vectors in 6 cell lines, respectively. CpG dinucleotides are represented by dark blue lollipops (filled: methylated CpG, empty: unmethylated CpG). First three CpGs are located within the promoter region, while the last three are located in the gene body. Gene expression or lack of it is represented by green arrows. **Right panel.** The yellow column shows methylation of a random CpG (used as a background), the methylation vector of this CpG demonstrates low correlation with the gene expression (the green box on the right, in RPKM). Correlation between the average promoter/gene body methylation (shown in the light blue and light purple columns, respectively) and the corresponding gene expression is also low. However, for the CpG TL (shown in the red box), the methylation significantly correlates with the gene expression

DNA methylation in promoter regions often repress gene expression. Nevertheless, the link between expression and promoter or gene body methylation is not straightforward, suggesting the need to deconvolute DNA methylation profiles into regulatory regions of a smaller size. To thoroughly investigate the connection between methylation and expression, we focus on methylation levels of single CpG dinucleotides. Following the logic previously reported in our works [40, 41], we expand our approach and use whole-genome DNA methylation (genome wide bisulfite sequencing, WGBS) and expression (RNA-seq) data for 48 normal human primary cells and tissues from the Roadmap Epigenomics Project. We selected non-related cell types to capture CpG position which are most variable in methylation between cell types. We define CpG traffic lights (CpG TL) as CpG dinucleotides with significant Spearman correlation coefficient (SCC) between DNA methylation and expression levels of a neighboring gene (*FDR*<0.01, Fig. 1).

Here we show that the average methylation of promoter/gene body less frequently correlates significantly with the gene expression compared to the methylation of CpG TL, even applying a proper multiple testing correction. In particular, at *FDR*<0.01 we find only 764/762 genes for which average promoter/gene body methylation correlates with expression, while at the same level of significance we observe 7997 genes correlating significantly with CpG TL methylation levels (Table 1, Additional file 1: Table S1, Table S2). Similar tendencies are observed for different promoter/gene body boundaries (Additional file 1: Table S3).

**Table 1:** The number of genes with significant correlation between expression and methylation

FDR-corrected *p*-value (significance level)	Total number of genes, which have significant correlations between gene expression and methylation			
	Average methylation of promoter regions (-1000..500) (1)	Average methylation of gene bodies (+500..TTS) (2)	Methylation of CpG TL (3)	Permutation test (4)
0.001	263	186	1463	14.5
0.005	537	505	4905	15.4
0.01	764	762	7997	16.2
0.05	2038	2125	22957	21.8
0.1	3251	3401	34095	27.5

Note: for multiple testing correction the number of genes was used in (1) and (2), while the number of all CpG - gene pairs was used for the same purpose in (3) and (4). (4) Permutation test (RPKM) results: the number of genes with significant correlation between expression and methylation obtained by chance (averaged over 10 random permutations). (TTS) refers to a Transcription Termination Site

The majority of promoter CpG TL demonstrate negative SCC, while the majority of those located in intronic regions demonstrate positive SCC, which is in line with the previous findings. Exonic CpG TL demonstrate comparable number of both positive and negative SCC with an increase in positive SCC towards gene 3’ end (Additional file 1: Figure S1). CpG TL are uniformly distributed along the genome (Manhattan plot, Additional file 1: Figure S2).

### CpG traffic lights are conserved across mammals and primates

**Fig. 2:**
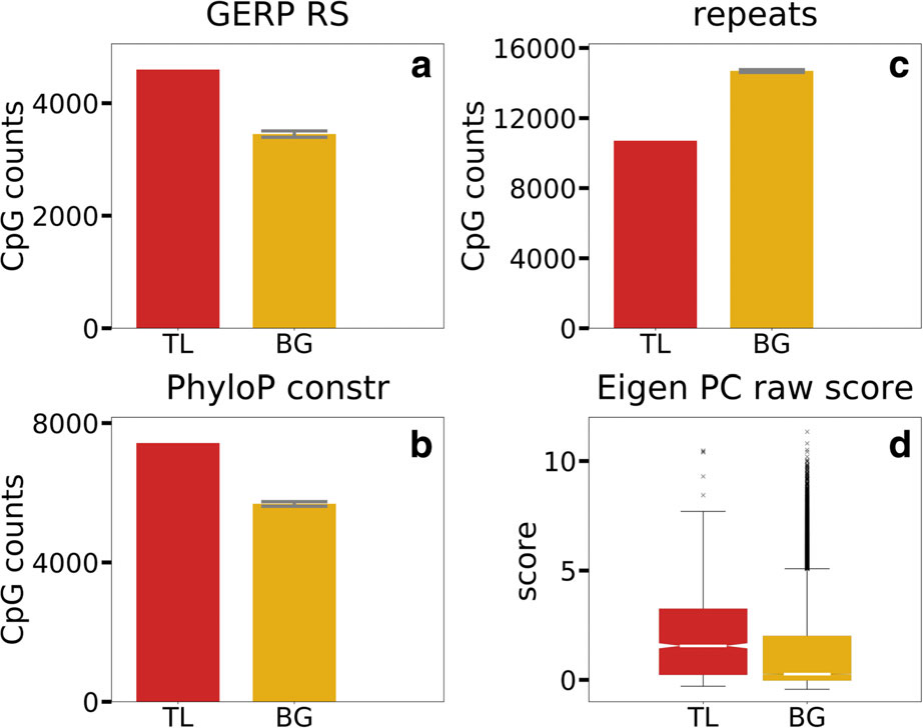
Evolutionary conservation of the CpG TL compared to the background CpG sites (BG). **a** Conservation in mammals and **b** in primates, **c** repeats determined by RepeatMasker, **d** Eigen non-coding functionality score. Whiskers (**abc**) represent standard deviation out of the 50 random background samples. Fisher exact test, *p*-value <5*E*−4 (**a** - **c**), Kolmogorov-Smirnov statistic for 2 samples *p*-value <5*E*−4 (**d**)

**Fig. 3:**
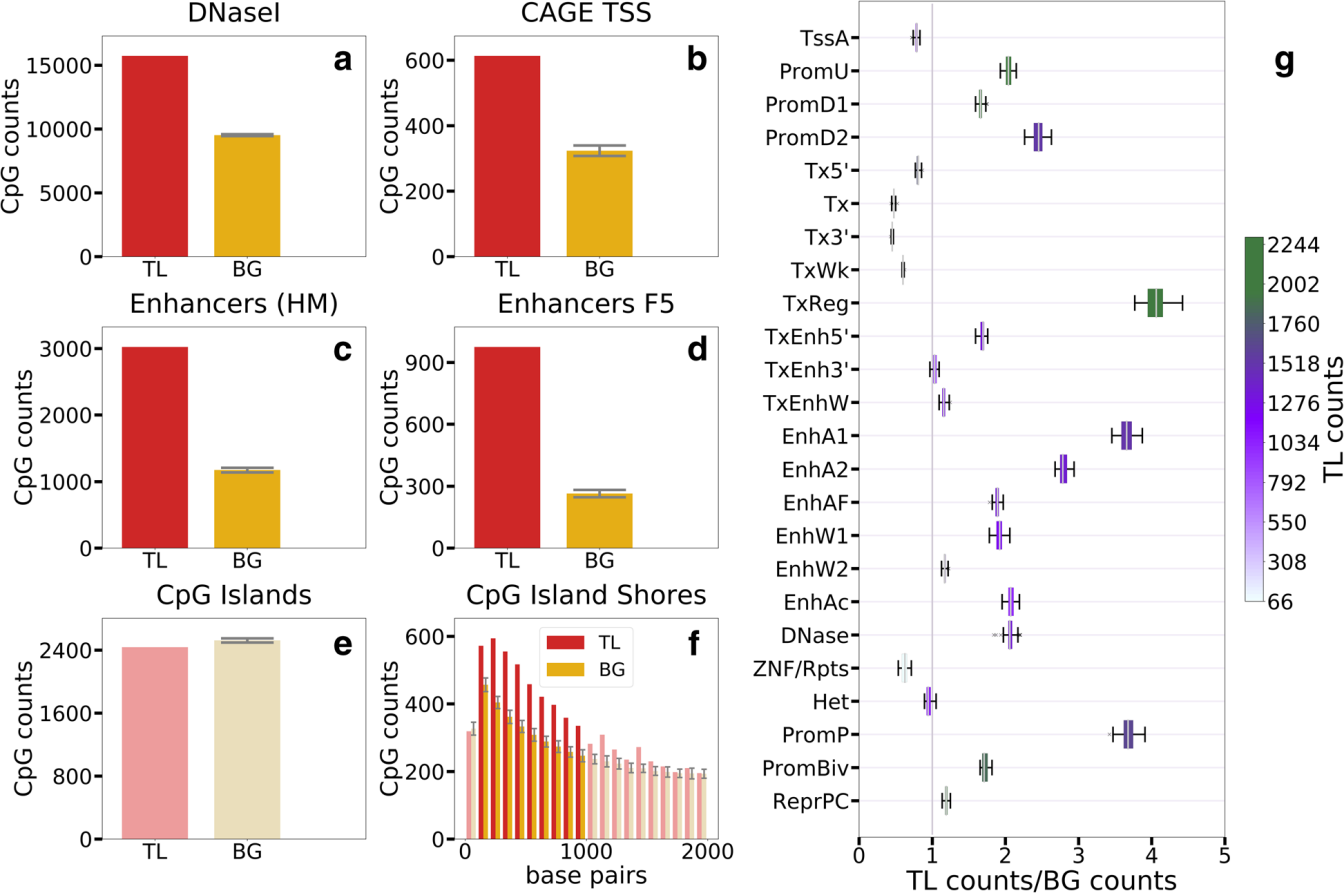
CpG TL in regulatory regions. Over-representation of CpG TL in **a** open chromatin regions (DNaseI), **b** transcription start sites determined by CAGE, **c** enhancers determined by histone modifications, **d** enhancers determined by FANTOM5. No difference between CpG TL and CpG BG counts in **e** CpG islands while **f** CpG TL are over-represented in CpG islands shores. Panel **g** represents averaged across 127 cell types ratio of TL / BG in chromatin states determined by chromHMM. The color **g** reflects absolute number of the CpG TL located in a given chromatin state. Whiskers (**a**-**f**) represent standard deviation of the 50 random background samples. Fisher exact test, *p*-value <5*E*−4

To address functionality of CpG TL, we first investigate their evolutionary conservation and find that CpG TL are preserved in mammals and in primates according to GERP RS [42] and PhyloP [43] scores respectively (Fig. 2a, b). Also, CpG TL are depleted in repetitive sequences determined by repeatMasker (Fig. 2c) (see “Methods”) and chromatin states (chromHMM [44], Fig. 3g). Eigen non-coding scores [45] that reflects non-coding functionality are significantly higher for CpG TL (Fig. 2d). Taken together, these results suggest the regulatory role of CpG TL in the genome.

### CpG traffic lights are enriched in regulatory elements

To narrow down the regulatory role of CpG TL we tested for the overlap between CpG TL and various functional genomic elements. CpG TL are enriched in the open chromatin regions (Fig. 3a) supporting the claim of their regulatory potential. In particular, they are 2-fold enriched at exact transcription start sites (Fig. 3b) determined by CAGE (Cap Analysis of Gene Expression) [46], as well as in all promoter types determined by chromHMM [44], including active, bivalent, and poised promoters but not in the regions of transcription elongation (Fig. 3g). Interestingly, the strongest enrichment was observed in poised promoters (>3.5 fold). Since the poised or bivalent chromatin is thought to be able to easily switch between active and repressed states [47], such enrichment may suggest a contribution of CpG TL to the maintenance of the bivalent state of the chromatin.

**Fig. 4:**
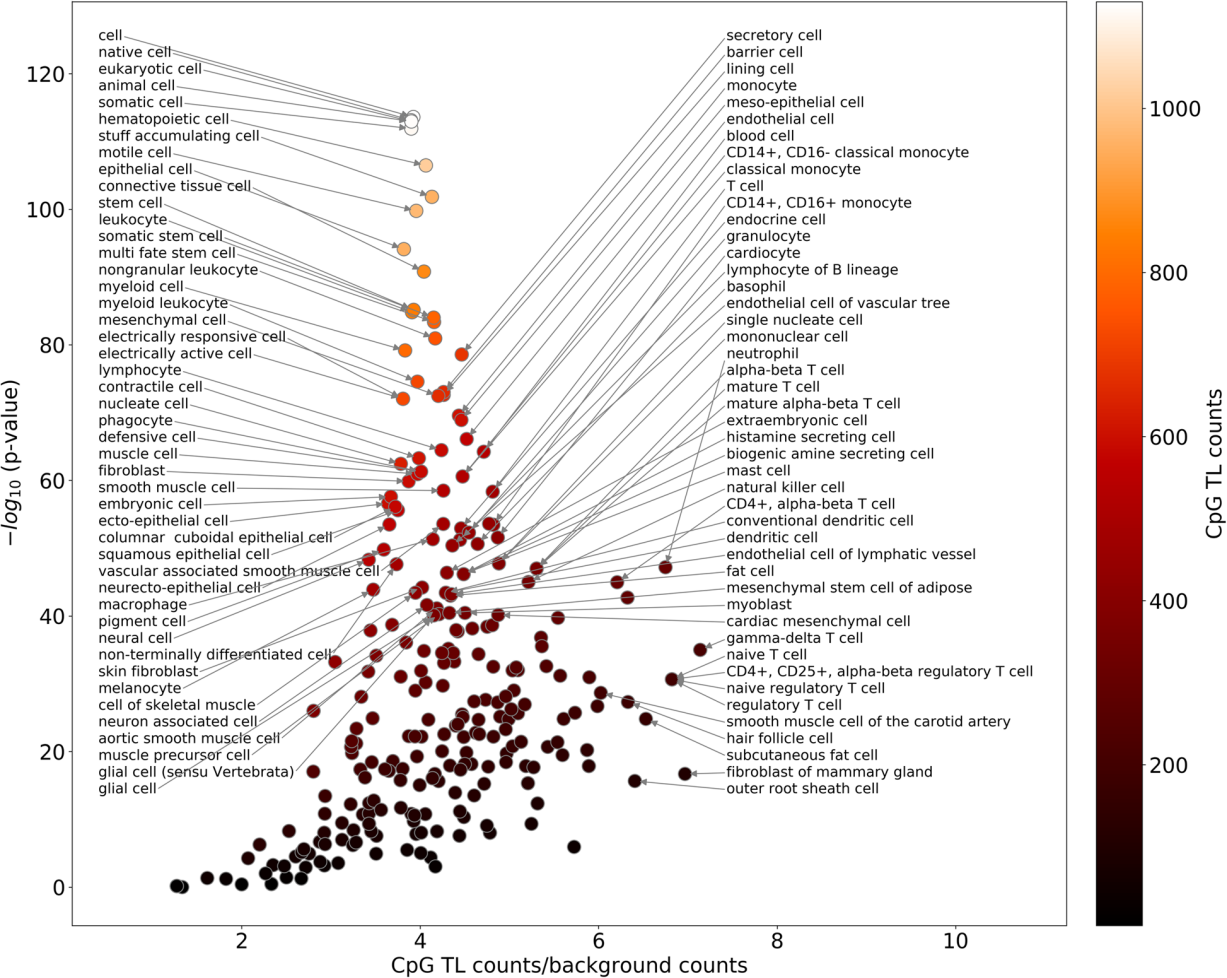
Functional categories of human enhancers enriched with CpG TL (negative SCC). Fisher’s exact test and FDR (Benjamini-Hochberg) correction for multiple testing (implemented in python scipy.stats.fisher_exact and p_adjust (method=’fdr’) from R) were used to calculate the *p*-values

CpG TL are also highly enriched in chromatin states corresponding to regulatory elements (Fig. 3c-g), in particular in enhancers, determined by a combination of histone marks (Fig. 3c), by CAGE bidirectional transcription (Fig. 3d), and by chromatin states (Fig. 3g). Among all enhancer types the most enriched are various stem cell and hematopoietic cell enhancers suggesting potential role of CpG TL methylation in regulation of pluripotency and hematopoesis (Fig. 4, Additional file 1: Table S4). Surprisingly, CpG TL are enriched in CpG island shores but not in CpG islands (Fig. 3e, f).

### CpG traffic lights are enriched in regulatory genes but avoid the majority of transcription factor binding sites

**Fig. 5:**
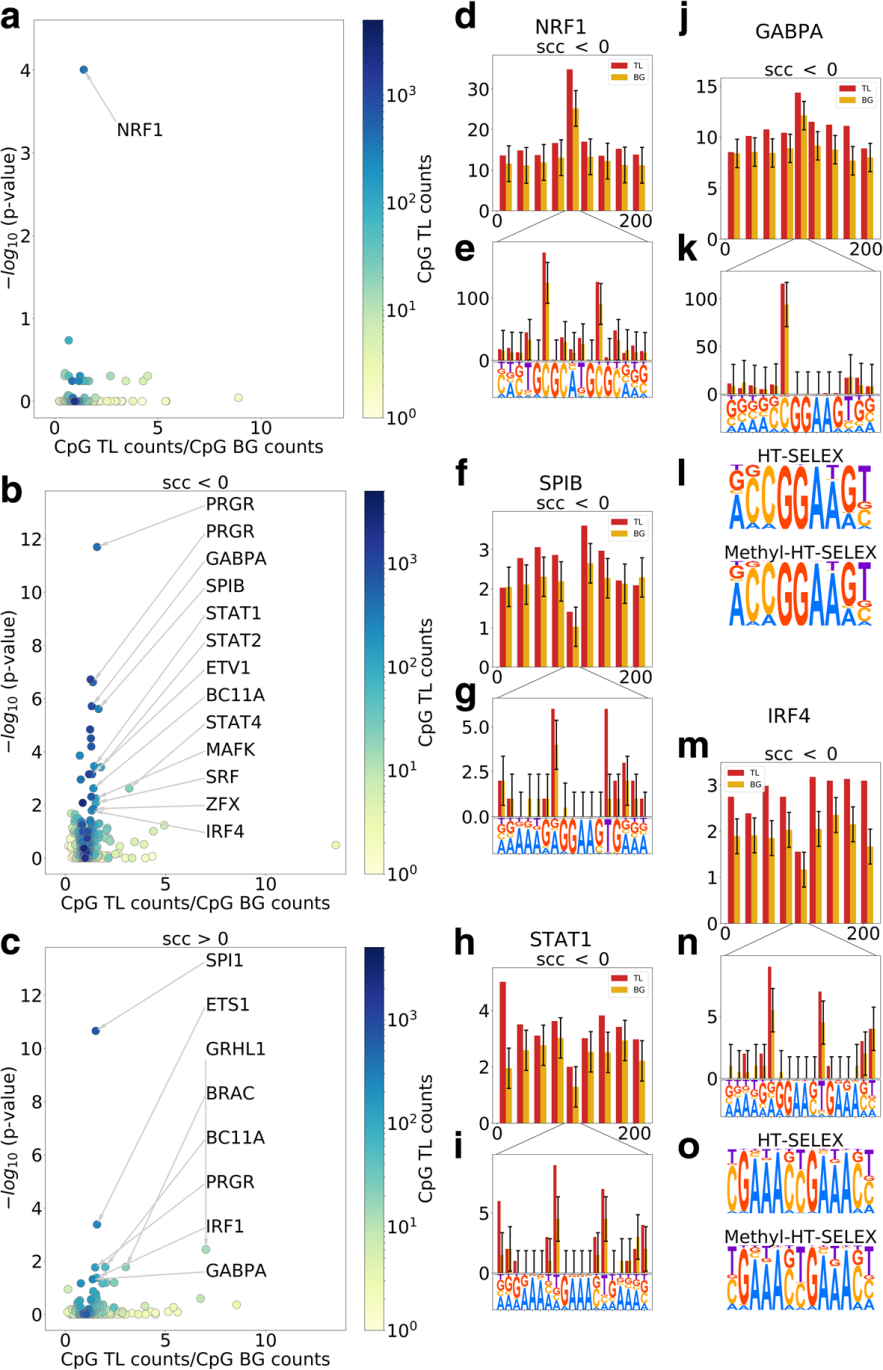
CpG TL within transcription factor binding sites. CpG TL to CpG BG ratio vs Fisher’s exact test *p*-value within **a** all predicted TFBS; **b** TFBS and 50 nt shores for CpG TL with a negative SCC and **c** the same for CpG TL with a positive SCC. Length-normalized distribution of CpG TL / CpG BG counts (negative SCC) within TFBS and 100 nt shores: **d** NRF1; **f** SPIB; **h** STAT1; **j** GABPA; **m** IRF4. Per position distribution of CpG TL / CpG BG counts (negative SCC) within TFBS with logo: **e** NRF1; **g** SPIB; **i** STAT1; **k** GABPA; **n** IRF4. In vitro binding preferences of unmethylated and methylated oligos: **l** GABPA; **o** IRF4

By analyzing the functionality of genes harboring CpG TLs we found a strong enrichment of such genes with known transcription regulators — transcription factors, co-factors and epigenetic regulators (Table 2, see “Methods”). Previously we reported that CpG TL avoided computationally predicted TFBS suggesting that direct methylation of TFBS may not be the main mechanism of TF binding regulation [40]. In this work, using aggregated data on in vivo binding (ChIP-Seq) we support this claim showing that for the majority of TFBS (with those of NRF1 being a notable exception) there is no enrichment for the CpG TL (Fig. 5a). Yet, surprisingly for some TF, CpG TL were enriched in the close proximity of their TFBS (Fig. 5b, c).

**Table 2:** Enrichment of CpG TL in regulatory genes

Gene type	# genes of in the annotation	# genes with CpG TL	# genes expected	fold enrichment	over-repre-senta-tion	*p*-value
Epigenetic regulators	719	279	98.56	2.83	+	1.4E-63
Histones	94	17	12.89	1.32	+	0.23
Transcription factors	1751	599	240.02	2.50	+	1.06E-108
Transcription co-factors	951	356	130.36	2.73	+	4.69E-76

#### NRF1 binding sites

Despite the observation that overall TFBS do not co-locate with CpG TL, binding sites of NRF1 (Fig. 5a, d) — a transcription factor involved into activation of key metabolic genes — are enriched in CpG TL even when overall enrichment for regulatory regions is taken into account (see “Methods”). Interestingly, core CpG positions of NRF1 binding sites are the most enriched with CpG TL supporting their functional importance for NRF1 binding (Fig. 5e). Being in line with the previous findings [48], these observations imply that NRF1 may be one of the very few TF whose binding may be directly regulated by DNA methylation.

#### ETS-family binding sites

Exact binding sites of GABPA (ETS-motif binding TF) and their close proximity (50bp) are 1.3-folds enriched in CpG TL (Fig. 5j, k). The strongest enrichment is observed in C neighboring the core GGAA box. In vitro binding data (HT-SELEX and Methyl-SELEX) [49] show that methylated C is less frequent in this position (Fig. 5l). Similar CpG TL enrichment was observed for binding sites of another members of ETS-familty: SPIB (Fig. 5f, g) and ETV1 (Additional file 1: Figure S3a-c). Binding of ETS-family members might be directly affected by DNA methylation, yet enrichment of the CpG TL in the closest proximity also supports the hypothesis of the indirect effect of regional methylation.

#### STAT-family and IRF-family binding sites

Surprisingly, such GA-rich motifs as those bound by STAT1,2,4 and IRF1,4 are also enriched in CpG TL but in their weak positions and in close proximity to the TFBS (Fig. 5h, i, m, n, Additional file 1: Figure S3d-k). In vitro binding data for IRF4 (HT-SELEX and Methyl-SELEX) shows an avoidance of methylated C in this motif position (Fig. 5o). Since the enrichment in CpG TL is observed only in weak motif positions we speculate that binding of the TF from STAT- and IRF-families is indirectly affected by methylation of the whole regulatory region.

## Discussion

In this work we demonstrate that methylation profiles of single CpG dinucleotides (CpG TL) more often significantly correlate with gene expression as compared to average promoter / gene body methylation. It is a surprising observation, since it is widely accepted that DNA methyltransferases once bound to DNA move along it [50] or multimerize [51] methylating all neighboring CpG dinucleotides unless a boundary protein, such as Sp1, is reached (reviewed in [52]). Yet, only a small fraction of CpG TL are co-located within the promoter (or body) of the same gene. We speculate that local change in DNA methylation could be achieved through active DNA demethylation probably with the help of TET proteins. A direct experiment with the use of CRISPR/TALEN-based technology is required to test this hypothesis.

It should be noted that our procedure of CpG TL detection based on correlation (SCC) cannot be applied to CpG dinucleotides that are fully methylated or methylated in all studied cell types. Our dataset consists of 48 cell types and does not cover the whole spectrum of human cell types. Due to this limitation, a significant fraction of regulatory CpG might be missing from our analysis. Novel data on DNA methylation and expression in various cell types will improve our understanding of CpG TL functions.

The enrichment of CpG TL in enhancers, in particular in hematopoietic enhancers, is in line with the recent reports that DNA methylatransferases DNMT3a/b can bind enhancers and regulate the enhancer RNA production in hematopoietic cells [22]. Also, distal regulatory regions can initiate transcription themselves, being in turn regulated by DNA methylation [53], contributing to the similarity of TSS and enhancers in terms of CpG TL enrichment.

Previously, it has been reported that NRF1 binding is directly regulated by DNA methylation [48]. In our work we demonstrate that such regulated binding is functional and regulate corresponding gene expression at least in some cases when NRF1 TFBS harbor a CpG TL. We also observed the enrichment of CpG TL in the close proximity to the ETS-, STAT- and IRF-family motifs hits. Interestingly, the majority of TF from these families are involved in hemapoietic regulation being in line with the strong enrichment of CpG TL in hematopoietic enhancers. These observations support the importance of the enhancer methylation in the regulation of the hematopoetic cells.

In the light of over-representation in regulatory regions, lack of enrichment of CpG TL within the majority of TFBS is puzzling. We can see several possible explanations. CpG TL may target unknown TFBS, although we believe that this scenario is unlikely. It was previously shown that almost all novel motifs obtained from regulatory regions correspond to known families of TFBS [46, 54, 55]. Furthermore, the HOCOMOCO v11 collection covers almost all structural families of transcription factors, except for the zinc finger family. Among those, there might be some important isolated cases enriched with CpG TL but their contribution to the overall picture is expected to be negligible. Alternatively, cytosine methylation could accumulate as a consequence of the absence of TF binding, which makes methylation of CpG TL not a primary cause, but just a “passive” marker of absent gene expression resulting from inactivation of its regulatory element. The last alternative is supported by previous works [56, 57]. More studies are needed to confirm which alternative is the most accurate. Yet, even if the “passive” marker explanation is true, CpG TL methylation could be a reliable marker of enhancer activity and gene expression, and can be used in practical applications, for example, in clinic where testing for DNA methylation is easier than testing directly for gene expression due to more stable nature of DNA.

## Conclusions

In this work we demonstrate that CpG TL are enriched in regulatory regions, including poised/bivalent promoters and enhancers, in particular in hematopoietic enhancers. Only a handful of TFBS, such as those bound by NRF1, could be directly regulated by DNA methylation, while binding of several TF families (ETS-, STAT-, IRF-) could be affected indirectly through methylation and repression of the entire regulatory region. CpG traffic lights provide a promising insight into gene regulation linking single CpG methylation to gene expression.

## Methods

### DNA methylation and expression data processing

We selected 48 tissues and cell types (see Additional file 1: Table S5) for which both WGBS and RNA-seq data were available in Roadmap Epigenomics Project. For all samples sequenced with the Illumina platform read trimming and adapter removal were performed by Trimmomatic [58] (up to 2 mismatches between an adapter and a read sequence; 5bp sliding window; quality threshold of 20; removing sequences shorter than 20 bp after trimming). For the samples sequenced with the SOLiD platform we used Cutadapt [59] (up to 10% error rate relative to the length of the matching region; quality threshold of 20; removing sequences shorter than 20 bp after trimming).

We mapped WGBS data to the genome (assembly GRCh38-Ensembl 78) with Bismark [60] (zero mismatches in the seed, 20bp seed length, 0/500bp the min/max insert size for valid paired-end alignments). Further we consider only methylated cytosines in the CpG context, covered with not less than 4 reads on both strands. For each CpG position in every of the 48 samples, the methylation values were averaged between replicates. We removed all CpG positions if methylation values were available for less than 20 samples.

We mapped RNA-Seq data with Tophat v2.0.13 [61] (up to 2 mismatches and 2 gaps per read, paired-end reads are reported only if both reads are mapped). We generated an expression matrix using FeatureCount [62], the expression profiles were normalized to RPKM values.

### CpG traffic lights detection

To determine CpG TL we considered all pairs of genes and CpG located within 10000 bp upstream of TSS to 3’ gene ends. One CpG might be associated with multiple genes, similarly, one gene might be associated with multiple CpG. For each CpG-gene pair we created two k-dimensional vectors (where k =20..48) of methylation levels (beta-values, [0,1]) and gene expression (RPKM). The length of the vectors (k) varies due to the fact that WGBS does not provide uniform coverage for all genomic CpG leading to missing values in the methylation profile of many CpGs. To avoid vague correlations we did not consider the CpG positions having less than 20 defined values in the respective methylation profiles. We further refer to each of the two vectors as a methylation and expression profiles. In total we had 18,830,232 CpGs associated with 59,396 genes (in total, 25,813,295 pairs).

For each CpG position, we calculated SCC between the methylation and expression profiles for all available samples. We refereed to a CpG position as a CpG traffic light (CpG TL) if it had a significant Spearman correlation coefficient (SCC) between methylation and expression profiles at the level of *FDR*<0.01 (Benjamini-Hochberg correction for the total number of pairs). We found 33,276 such CpG TL (0.18% of the original number of CpGs) that corresponded to 7997 genes.

### Construction of background datasets

To explore enrichment of CpG TL within various genomic regions we constructed background sets (CpG BG) of the same size. We required CpG BG to be similar to CpG TL based on the following criteria:

- GC content (the total number of C and G nucleotides) of the surrounding region of CpG BG must be similar to that of CpG TL. We calculated GC content in 200 bp windows centered on each CpG TL. For each such TL-centered window, we searched for another genomic CpG with the surrounding window having no more than 5% difference in GC content. For example, if there are 80 cytosines and guanines in a 200 bp window around CpG TL, we were looking for a CpG BG having from 76 to 84 cytosines and guanines in a 200 bp window.
- CpG content (the total number of CG pairs) of the surrounding region of CpG BG should be similar to that of CpG TL. Again, for each CpG TL we allowed no more than 5% difference in CpG content in a 200 bp window.
- CpG BG should have a similar distance to the TSS of the associated gene (while accounting for upstream or downstream location). For this purpose, we separately considered CpG TLs in [−100;*TSS*] and [*TSS*;100] distance bins by collecting CpG BG from the respective regions. For CpG TLs located farther than 100 bp from TSS, we considered *log*_10_(*distance*) and allowed up to 5% difference between CpG TL and its respective CpG BG. E.g. if a CpG TL is located + 1000 from a TSS, we are looking for a CpG BG located [708;1413].

A background CpG for a CpG TL with a *SCC*<0 (*SCC*>0) should also have a negative (positive) SCC with at least one of the associated genes). We repeated the selection process 50 times.

It is important to note that we did not control for the presence of a CpG island (CGI). Recently it has been shown that even methylated CpG dinucleotides within CpG islands were more conserved in primate evolution compared to methylated CpG outside the CGI [63]. Yet algorithms for CGI search use arbitrary parameters and may not be accurate in determination of CGI boundaries [64]. Therefore, controlling for a presence of a CGI would not necessarily reduce this bias.

### Genomic annotations

We annotated all CpG positions with overlapping genomic features. For each feature we calculated the over-representation of CpG TL over CpG BG within each annotation using the exact Fisher’s test (in the total number of CpG TL and for CpG TL with positive/negative SCC separately). The following genomic annotations were tested: repeats (RepeatMasker http://hgdownload.soe.ucsc.edu/goldenPath/hg38/database/rmsk.txt.gz); the robust CAGE clusters [46]; the robust enhancers [65] (mapped to hg38 with the liftOver); the DNaseI hypersensitivity clusters (http://hgdownload.soe.ucsc.edu/goldenPath/hg38/database/wgEncodeRegDnaseClustered.txt.gz). Functional annotation of the enhancers was obtained from [46, 54, 55].

#### Evolutionary conservation and Eigen scores

Conservation of CpG TL and background sites in mammals and primates was assessed with UCSC Genome Browser GERP RS [42] and PhyloP [43] hg19 tracks, respectively. We calculated how many sites in each dataset had GERP RS score greater than 2, which we considered as conserved in mammals and PhyloP score greater than 0.5, which we considered as conserved in primates. Overall functional scores for each site were calculated with Eigen [45]. Higher Eigen scores imply more likely functionality of respective genome sites.

#### Histone modifications and chromatin states

The Roadmap Epigenomics Consortium 25-state segmentation of 127 epigenomes predicted with ChromHMM [44, 66] was used to assess chromatin states co-located with CpG TL. The annotation based on the imputed data for 12 chromatin marks (H3K4me1, H3K4me2, H3K4me3, H3K9ac, H3K27ac, H4K20me1, H3K79me2, H3K36me3, H3K9me3, H3K27me3, H2A.Z, and DNaseI) was downloaded from http://egg2.wustl.edu/roadmap/web_portal/imputed.html#chr_imp. We calculated a CpG TL/CpG BG ratio for each of the 25 chromatin states in each of the 127 epigenomes and then averaged the ratios for a representation on a figure.

Additionally, to verify CpG TL enrichment in the enhancers we selected regions having H3K27ac and H3K4me1 but lacking H3K4me3 (ENCODE, averaged among all samples mapped to hg38 with pre-calculated narrowPeak available, files with major errors and warnings excluded) (Additional file 1: Table S6).

#### TFBS prediction

For transcription factor binding sites prediction, we used position weight matrices (PWM) of human TFs provided in full HOCOMOCO v11 [67] collection and its default PWM thresholds according to the pre-calculated motif *P*-value of 0.0005 as in [68]. In HOCOMOCO v11, the thresholds and *P*-value were estimated against whole-genome dinucleotide composition. However, prediction of TFBS using PWMs alone can result in a notable number of false positives. Having this in mind, out of all predicted TFBS, we considered only those located in the reproducible and control data-supported cistrome [69] (only A, B, and C cistrome categories) for each TF. The cistrome was constructed from the ChIP-Seq data on transcription factors provided in the GTRD database [70] and processed by a common pipeline involving several computational ChIP-Seq peak callers, allowing to capture binding events routinely detected in different experiments. Thus, the TFBS considered in our study, were supported both by computational sequence analysis and by experimental ChIP-Seq data.

#### Gene enrichment analysis

We tested if genes that harbor CpG TL were enriched in transcription factors, co-factors and epigenetic regulators using Fisher’s exact test (implemented in python library scipy.stats) with Bonferroni correction. A list of TF and co-TF was obtained from Tcof DB [71] and the list of epigenetic regulators was obtained from EpiFactors [72].

## Additional file

Additional file 1 Supplementary materials. **Figure S1:** SCC of the CpG TL located in various gene regions; **Figure S2:** Distribution of CpG TLs along the genome; **Figure S3:** TFBS; **Table S1:** Number of significant SCC between average methylation of genomic region and gene expression; **Table S2:** Number of significant SCC between CpG methylation and gene expression; **Table S3:** Number of significant SCC between gene expression and average methylation of the genome region; **Table S4:** Most enriched with CpG TLs categories of enhancers; **Table S5:** Names of the cell samples in the study; **Table S6:** Enhancers = H3K27ac+H3K4me1-H3K4me3; **Table S7:** Expression data source; **Table S8:** Methylation data source. (PDF 2562 kb)

## Abbreviations

CAGE: Cap analysis of gene expression
CGI: CpG island
ChIP-Seq: Chromatin immunoprecipitation (ChIP) sequencing
CpG: CpG dinucleotide, 5’—C—phosphate—G—3’
CpG BG: background CpG dinucleotide
CpG TL: CpG traffic light
ENCODE: Encyclopedia of DNA elements
FANTOM5: Functional annotation of mammalian genome 5
FDR: False discovery rate
PWM: Position weight matrice
RNA-seq: RNA sequencing
RPKM: Reads per kilobase per million
SCC: Spearman correlation coefficient
TF: Transcription factor
TFBS: Transcription factor binding sites
TSS: Transcription start sites
WGBS: genome wide bisulfite sequencing
