# Supplementary material for: CpG traffic lights are markers of regulatory regions in human genome
Source: BMC Genomics. 2019 Feb 1;20:102. doi: 10.1186/s12864-018-5387-1 (PMC6359853; doi:10.1186/s12864-018-5387-1)
Supplement: Supplementary file 1 — Supplementary materials. Figure S1: SCC of the CpG TL located in various gene regions; Figure S2: Distribution of CpG TLs along the genome; Figure S3: TFBS; Table S1: Number of significant SCC between average methylation of genomic region and gene expression; Table S2: Number of significant SCC between CpG methylation and gene expression; Table S3: Number of significant SCC between gene expression and average methylation of the genome region; Table S4: Most enriched with CpG TLs categories of enhancers; Table S5: Names of the cell samples in the study; Table S6: Enhancers = H3K27ac+H3K4me1-H3K4me3; Table S7: Expression data source; Table S8: Methylation data source. (PDF 2562 kb) [file 12864_2018_5387_MOESM1_ESM.pdf]

Supplementary materials

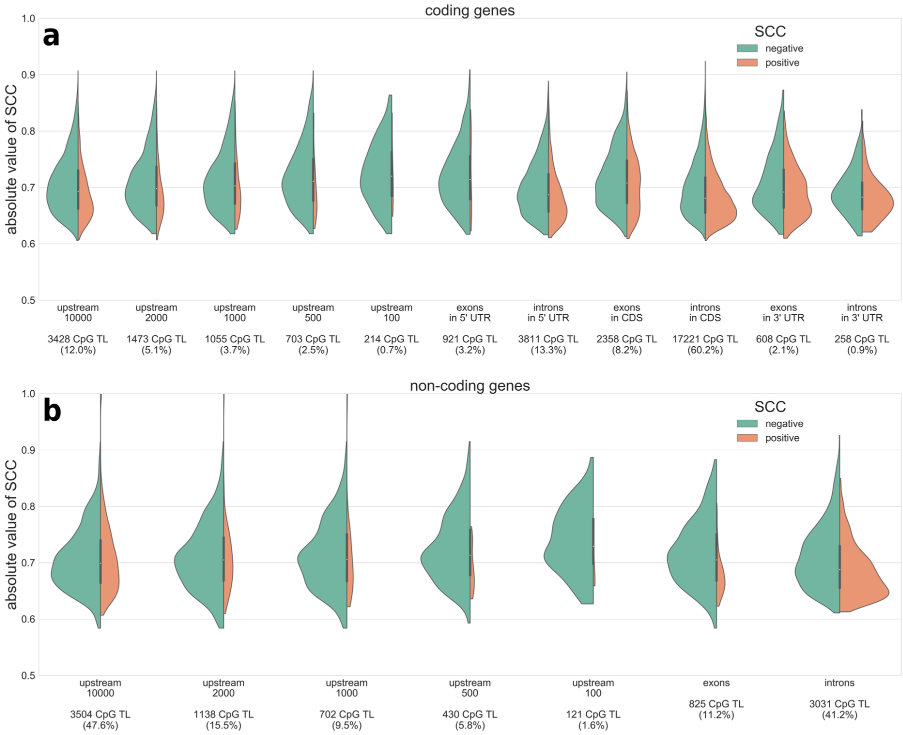

Supplementary Figure S1. SCC of the CpG TL located in various gene regions The total number of CpG TL in promoters, exons and introns are presented at the bottom. Green (left) / pink (right) parts of the violin plots show the distribution of positive and negative SCC, respectively. (a) protein coding genes, (b) non coding genes

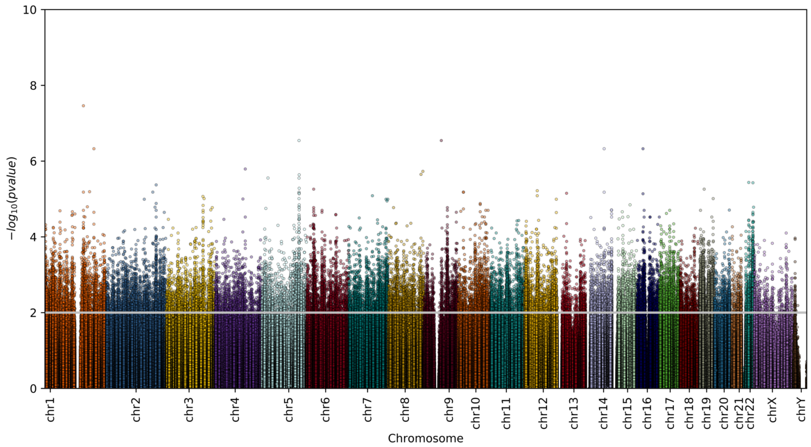

Supplementary Figure S2. Distribution of CpG TLs along the genome.

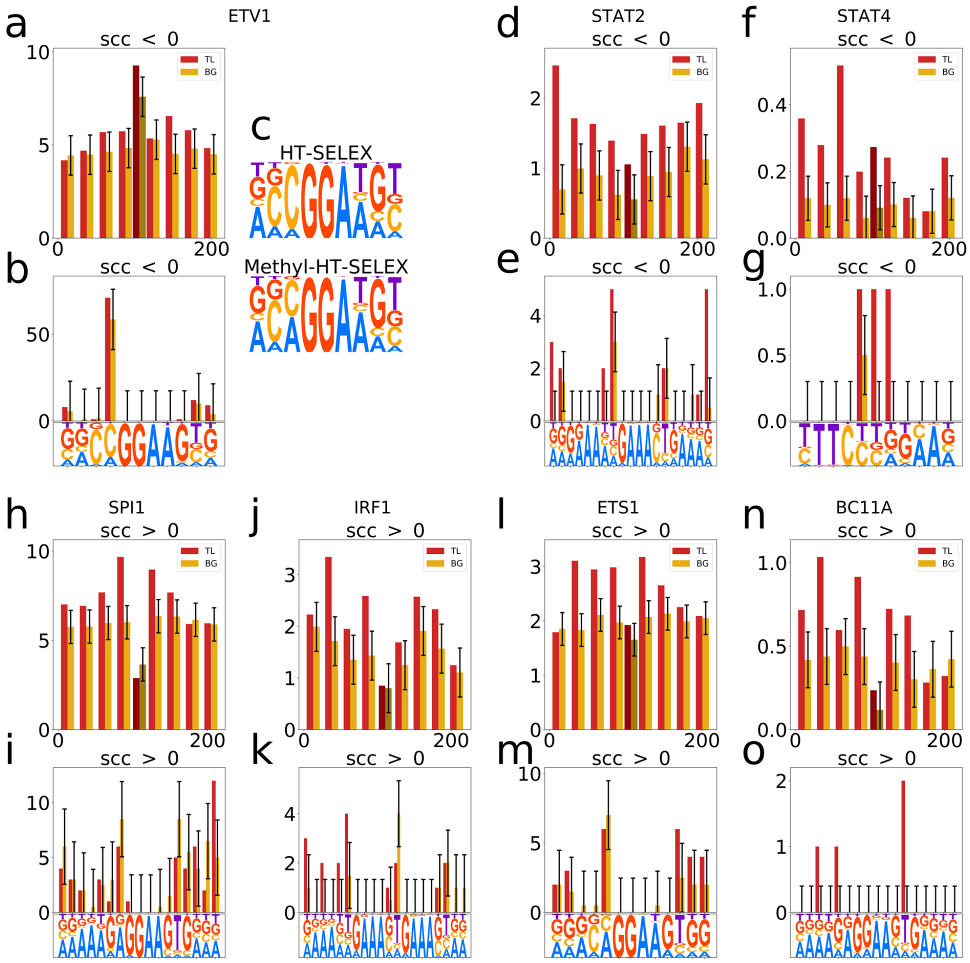

Supplementary Figure S3. TFBS

Supplementary Table S1. Number of significant SCC between average methylation of genomic region and gene expression. Permutation test (10 random shuffles of expression) compared to original data. Multiple testing correction was performed using Benjamini Hochberg FDR correction. Total number of genes = 59,396

FDR	number (averaged over 10 random shuffles) of genes with significant SCC between average promoter (-1000 ... +500) methylation and expression	number of genes with significant SCC between average promoter (-1000 ... +500) methylation and expression	fraction of genes number with significant SCC between average promoter (-1000 ... +500) methylation and expression	number (averaged over 10 random shuffles) of genes with significant SCC between average gene body (+500 ... TTS) methylation and expression	number of genes with significant SCC between average gene body (+500 ... TTS) methylation and expression	fraction of genes number with significant SCC between average gene body (+500 ... TTS) methylation and expression
0.0001	0.0 ± 0	95	1.6e-03	0.0 ± 0	61	1.0e-03
0.0002	0.0 ± 0	115	1.9e-03	0.0 ± 0	83	1.4e-03
0.0003	0.0 ± 0	140	2.4e-03	0.0 ± 0	103	1.7e-03
0.0004	0.0 ± 0	166	2.8e-03	0.0 ± 0	126	2.1e-03
0.0005	0.0 ± 0	188	3.2e-03	0.0 ± 0	136	2.3e-03
0.0006	0.0 ± 0	213	3.6e-03	0.0 ± 0	145	2.4e-03
0.0007	0.0 ± 0	227	3.8e-03	0.0 ± 0	157	2.6e-03
0.0008	0.0 ± 0	238	4.0e-03	0.0 ± 0	168	2.8e-03
0.0009	0.0 ± 0	248	4.2e-03	0.0 ± 0	174	2.9e-03
0.001	0.0 ± 0	263	4.4e-03	0.0 ± 0	186	3.1e-03
0.002	0.0 ± 0	367	6.2e-03	0.1 ± 0	284	4.8e-03
0.003	0.0 ± 0	445	7.5e-03	0.1 ± 0	357	6.0e-03
0.004	0.0 ± 0	488	8.2e-03	0.1 ± 0	445	7.5e-03
0.005	0.0 ± 0	537	9.0e-03	0.1 ± 0	505	8.5e-03
0.006	0.0 ± 0	585	9.8e-03	0.1 ± 0	563	9.5e-03
0.007	0.0 ± 0	629	1.1e-02	0.1 ± 0	608	1.0e-02
0.008	0.0 ± 0	669	1.1e-02	0.1 ± 0	641	1.1e-02
0.009	0.0 ± 0	712	1.2e-02	0.1 ± 0	703	1.2e-02
0.01	0.0 ± 0	764	1.3e-02	0.1 ± 0	762	1.3e-02
0.02	0.0 ± 0	1133	1.9e-02	0.1 ± 0	1167	2.0e-02
0.03	0.0 ± 0	1473	2.5e-02	0.1 ± 0	1486	2.5e-02
0.04	0.0 ± 0	1761	3.0e-02	0.2 ± 0	1772	3.0e-02
0.05	0.0 ± 0	2038	3.4e-02	0.2 ± 0	2125	3.6e-02
0.06	0.0 ± 0	2327	3.9e-02	0.2 ± 0	2349	4.0e-02
0.07	0.0 ± 0	2546	4.3e-02	0.2 ± 0	2618	4.4e-02
0.08	0.1 ± 0	2783	4.7e-02	0.2 ± 0	2857	4.8e-02
0.09	0.1 ± 0	3008	5.1e-02	0.2 ± 0	3122	5.3e-02
0.1	0.1 ± 0	3251	5.5e-02	0.2 ± 0	3401	5.7e-02
0.2	0.4 ± 1	6087	1.0e-01	0.2 ± 0	5941	1.0e-01
0.3	0.4 ± 1	9424	1.6e-01	0.3 ± 1	8761	1.5e-01
0.4	1.3 ± 2	13023	2.2e-01	1.0 ± 2	11667	2.0e-01
0.5	3.4 ± 3	17901	3.0e-01	2.1 ± 3	15063	2.5e-01
0.6	6.2 ± 5	22907	3.9e-01	5.0 ± 6	18932	3.2e-01
0.7	9.7 ± 9	28940	4.9e-01	13.1 ± 15	22993	3.9e-01

0.8	30.0 ± 27	35739	6.0e-01	39.0 ± 34	27577	4.6e-01
0.9	109.0 ± 116	44164	7.4e-01	138.1 ± 120	33274	5.6e-01
1.0	6651.9 ± 9652	54828	9.2e-01	13166.3 ± 12753	40835	6.9e-01

Supplementary Table S2. Number of significant SCC between CpG methylation and gene expression. Permutation test (10 random shuffles of expression) compared to original data. Multiple testing correction was performed using Benjamini Hochberg FDR correction. Total number of CpG gene pairs = 25,813,295

FDR	number (averaged over 10 random shuffles) of CpG-gene pairs with significant SCC between methylation and expression	number of CpG-gene pairs with significant SCC	fraction of CpG-gene pairs number (averaged over 10 random shuffles) with significant SCC between methylation and expression	fraction of CpG-gene pairs number with significant SCC between methylation and expression	number of genes (averaged over 10 random shuffles) with at least one significant SCC between any close CpG ans gene expression	number of genes with at least one significant SCC between any close CpG ans gene expression
0.0001	19.2 ± 5	340	7.4e-07	1.3e-05	13.6 ± 2	196
0.0002	19.3 ± 6	714	7.5e-07	2.8e-05	13.7 ± 3	375
0.0003	19.4 ± 5	1117	7.5e-07	4.3e-05	13.8 ± 3	527
0.0004	19.5 ± 5	1491	7.6e-07	5.8e-05	13.9 ± 3	671
0.0005	19.7 ± 6	1882	7.6e-07	7.3e-05	14.1 ± 3	817
0.0006	19.8 ± 6	2325	7.7e-07	9.0e-05	14.2 ± 3	974
0.0007	19.8 ± 6	2712	7.7e-07	1.1e-04	14.2 ± 3	1113
0.0008	19.8 ± 6	3104	7.7e-07	1.2e-04	14.2 ± 3	1244
0.0009	20.0 ± 6	3452	7.7e-07	1.3e-04	14.4 ± 3	1360
0.001	20.1 ± 6	3774	7.8e-07	1.5e-04	14.5 ± 3	1463
0.002	20.5 ± 6	7754	7.9e-07	3.0e-04	14.9 ± 4	2524
0.003	20.8 ± 7	11290	8.1e-07	4.4e-04	15.1 ± 4	3361
0.004	20.9 ± 7	14888	8.1e-07	5.8e-04	15.2 ± 4	4152
0.005	21.1 ± 7	18377	8.2e-07	7.1e-04	15.4 ± 4	4905
0.006	21.4 ± 7	22172	8.3e-07	8.6e-04	15.6 ± 4	5623
0.007	21.5 ± 7	25670	8.3e-07	9.9e-04	15.7 ± 4	6263
0.008	21.5 ± 7	29116	8.3e-07	1.1e-03	15.7 ± 4	6901
0.009	21.6 ± 7	32538	8.4e-07	1.3e-03	15.8 ± 4	7474
0.01	22.0 ± 6	35918	8.5e-07	1.4e-03	16.2 ± 4	7997
0.02	24.1 ± 7	71741	9.3e-07	2.8e-03	18.2 ± 4	12852
0.03	26.1 ± 7	109005	1.0e-06	4.2e-03	20.1 ± 4	16604
0.04	26.9 ± 8	148339	1.0e-06	5.7e-03	20.9 ± 5	19930
0.05	27.8 ± 8	189990	1.1e-06	7.4e-03	21.8 ± 5	22957
0.06	28.2 ± 7	232923	1.1e-06	9.0e-03	22.2 ± 5	25629

0.07	29.9 ± 8	278708	1.2e-06	1.1e-02	23.8 ± 6	28117
0.08	30.8 ± 9	325107	1.2e-06	1.3e-02	24.7 ± 6	30263
0.09	32.6 ± 9	374732	1.3e-06	1.5e-02	26.4 ± 6	32304
0.1	33.7 ± 9	425413	1.3e-06	1.6e-02	27.5 ± 6	34095
0.2	50.7 ± 16	1056473	2.0e-06	4.1e-02	43.6 ± 13	46819
0.3	81.0 ± 34	1935953	3.1e-06	7.5e-02	72.4 ± 26	52918
0.4	149.3 ± 66	3126752	5.8e-06	1.2e-01	135.7 ± 55	55670
0.5	310.7 ± 103	4671655	1.2e-05	1.8e-01	281.8 ± 86	56966
0.6	761.8 ± 121	6896109	3.0e-05	2.7e-01	673.6 ± 97	57953
0.7	1882.3 ± 431	9648189	7.3e-05	3.7e-01	1564.3 ± 324	58123
0.8	5544.2 ± 1276	13160124	2.1e-04	5.1e-01	4046.6 ± 735	58195
0.9	19904.1 ± 4895	17783535	7.7e-04	6.9e-01	10938.0 ± 1750	58240

Supplementary Table S3. Number of significant SCC between gene expression and average methylation of the genome region

region	0.001	0.005	0.01	0.05	0.1
-10000...TSS	46	159	284	1246	2376
-10000...+50	48	163	284	1206	2336
-10000...+100	56	159	275	1169	2269
-10000...+200	55	163	265	1130	2252
-10000...+300	52	155	247	1102	2176
-10000...+500	55	163	261	1100	2179
-5000...TSS	64	224	335	1170	2174
-5000...+50	79	225	327	1107	2151
-5000...+100	78	230	323	1135	2134
-5000...+200	80	234	321	1150	2197
-5000...+300	88	233	307	1202	2217
-5000...+500	94	237	337	1232	2289
-2000...TSS	124	298	424	1405	2457
-2000...+50	134	313	479	1432	2446
-2000...+100	148	321	489	1441	2465
-2000...+200	137	354	545	1509	2538
-2000...+300	147	380	568	1553	2620
-2000...+500	179	429	618	1713	2738
-1000...TSS	132	339	535	1497	2629
-1000...+50	166	384	570	1573	2771
-1000...+100	168	406	573	1644	2802
-1000...+200	194	428	630	1733	2893
-1000...+300	200	462	686	1819	3072
-1000...+500	263	537	764	2038	3251
-500...TSS	146	344	500	1392	2408
-500...+50	160	383	548	1497	2599
-500...+100	166	413	585	1581	2710

-500...+200	214	450	634	1743	2889
-500...+300	229	487	685	1830	3016
-500...+500	292	586	834	2076	3431
-400...TSS	131	330	460	1322	2251
-400...+50	162	372	508	1394	2486
-400...+100	178	418	550	1462	2553
-400...+200	220	469	611	1633	2782
-400...+300	239	508	696	1784	2983
-400...+500	302	601	818	2076	3366
-300...TSS	143	288	415	1204	2028
-300...+50	154	352	465	1306	2213
-300...+100	169	396	524	1396	2352
-300...+200	197	456	590	1537	2569
-300...+300	229	494	679	1682	2788
-300...+500	279	565	790	2004	3274
-200...TSS	127	238	342	976	1684
-200...+50	142	302	413	1134	1883
-200...+100	167	337	469	1224	2034
-200...+200	209	411	551	1412	2320
-200...+300	228	459	619	1586	2562
-200...+500	269	547	726	1789	3176
-100...TSS	86	168	246	684	1176
-100...+50	114	239	322	903	1586
-100...+100	141	282	377	1021	1815
-100...+200	170	352	472	1264	2041
-100...+300	185	398	550	1377	2294
-100...+500	233	513	675	1673	2791
-50...TSS	51	91	143	445	843
-50...+50	90	183	268	756	1267
-50...+100	115	248	334	943	1562
-50...+200	145	323	467	1146	1927
-50...+300	183	374	537	1269	2166
-50...+500	241	499	676	1586	2696
TSS...+50	47	129	193	585	940
TSS...+100	85	191	281	783	1328
TSS...+200	123	287	417	1048	1749
TSS...+300	146	341	485	1221	1992
TSS...+500	226	454	603	1476	2511
+0...TTS	159	390	581	1704	2944
+100...TTS	173	415	591	1822	3042
+200...TTS	176	412	626	1855	3130
+300...TTS	184	466	670	1940	3186
+400...TTS	188	483	722	2013	3266
+500...TTS	186	505	762	2125	3401
+1000...TTS	201	565	818	2321	3741

Supplementary Table S4. **Most enriched with CpG TLs categories of enhancers**

Enhancer category	CpG TL/BG ratio	P value
cell	3.923	2.532E-114
native cell	3.897	7.417E-114
eukaryotic cell	3.901	1.128E-113
animal cell	3.901	1.128E-113
somatic cell	3.900	1.247E-112
hematopoietic cell	4.064	3.409E-107
stuff accumulating cell	4.132	1.478E-102
motile cell	3.956	1.726E-100
epithelial cell	3.817	7.719E-95
connective tissue cell	4.042	1.568E-91
stem cell	3.927	6.912E-86
leukocyte	3.909	1.542E-85
somatic stem cell	4.151	9.780E-85
multi fate stem cell	4.156	4.258E-84
nongranular leukocyte	4.169	1.124E-81
myeloid cell	3.831	6.720E-80
secretory cell	4.466	2.672E-79
myeloid leukocyte	3.969	2.746E-75
barrier cell	4.263	9.351E-74
lining cell	4.263	2.437E-73
mesenchymal cell	4.203	3.383E-73
electrically responsive cell	3.808	9.240E-73
electrically active cell	3.808	9.240E-73
monocyte	4.434	2.806E-70
meso-epithelial cell	4.462	1.225E-69
endothelial cell	4.521	7.970E-67
lymphocyte	4.238	3.485E-65
blood cell	4.713	5.952E-65
contractile cell	3.985	5.423E-64
nucleate cell	3.783	3.616E-63
phagocyte	4.010	5.099E-62
defensive cell	4.010	5.099E-62
muscle cell	3.971	1.166E-61
CD14+, CD16- classical monocyte	4.475	2.631E-61
classical monocyte	4.475	2.631E-61
fibroblast	3.871	1.393E-60
smooth muscle cell	4.260	3.254E-59
T cell	4.813	4.617E-59
embryonic cell	3.672	2.541E-58
ecto-epithelial cell	3.640	2.461E-57
columnar cuboidal epithelial cell	3.722	7.650E-57
squamous epithelial cell	3.751	2.517E-56
vascular associated smooth muscle cell	4.259	2.663E-54
neurecto-epithelial cell	3.655	3.242E-54
CD14+, CD16+ monocyte	4.820	3.511E-54
endocrine cell	4.459	1.192E-53
granulocyte	4.547	5.093E-53

cardiocyte	4.870	2.729E-52
macrophage	4.142	4.737E-52
lymphocyte of B lineage	4.447	5.886E-52
basophil	4.644	2.376E-51
endothelial cell of vascular tree	4.361	4.079E-51
pigment cell	3.592	1.605E-50
neural cell	3.424	5.124E-49
single nucleate cell	4.882	1.786E-48
mononuclear cell	4.882	1.786E-48
non-terminally differentiated cell	3.735	2.339E-48
neutrophil	6.749	6.521E-48
alpha-beta T cell	5.305	1.021E-47
mature T cell	5.305	1.021E-47
mature alpha-beta T cell	5.305	1.021E-47
extraembryonic cell	4.299	4.213E-47
histamine secreting cell	4.487	6.807E-47
biogenic amine secreting cell	4.487	6.807E-47
mast cell	4.487	6.807E-47
natural killer cell	5.214	9.855E-46
CD4+, alpha-beta T cell	6.208	1.058E-45
skin fibroblast	4.022	6.369E-45
melanocyte	3.474	1.306E-44
conventional dendritic cell	4.288	3.765E-44
cell of skeletal muscle	3.948	3.765E-44
dendritic cell	4.288	3.765E-44
endothelial cell of lymphatic vessel	4.351	6.258E-44
fat cell	4.340	1.010E-43
neuron associated cell	4.076	2.456E-42
aortic smooth muscle cell	4.187	7.547E-42
mesenchymal stem cell of adipose	4.327	3.557E-41
myoblast	4.502	3.557E-41
muscle precursor cell	4.212	5.407E-41
cardiac mesenchymal cell	4.875	7.126E-41
glial cell (sensu Vertebrata)	4.153	8.093E-41
glial cell	4.153	8.093E-41
gamma-delta T cell	7.138	1.004E-35
naive T cell	6.821	2.246E-31
CD4+, CD25+, alpha-beta regulatory T cell	6.821	2.246E-31
naive regulatory T cell	6.821	2.246E-31
regulatory T cell	6.821	2.246E-31
smooth muscle cell of the carotid artery	6.024	2.211E-29
hair follicle cell	6.329	4.923E-28
subcutaneous fat cell	6.529	1.533E-25
fibroblast of mammary gland	6.968	2.002E-17
outer root sheath cell	6.405	2.234E-16

Supplementary Table S5. **Names of the cell samples in the study**

Sample Name	Number of Methylation Replicates	Number of Expression Replicates
adipose	3	3
adrenal gland	3	3
bladder	3	1
Brain Germinal Matrix	1	1
Breast Luminal Epithelial Cells	1	3
Breast Myoepithelial Cells	3	3
CD14 primary cells	3	1
CD3 primary cells	3	2
CD56 primary cells	3	1
esophagus	3	3
gastric	3	3
H1 +BMP4 cell line	3	2
H1 BMP4 derived mesendoderm cultured cells	3	2
H1 cell line	3	3
H1 derived mesenchymal stem cells	3	2
H1 derived neuronal progenitor cultured cells	3	3
H9 cell line	3	1
heart aorta	3	3
heart left ventricle	3	3
heart right atrium	3	3
heart right ventricle	3	3
hESC-derived CD184+ endoderm cultured cells	3	3
hESC-derived CD56+ ectoderm cultured cells	3	3
hESC-derived CD56+ mesoderm cultured cells	3	3
HUES64 cell line	3	3
IMR90 cell line	3	3
iPS DF 19.11 cell line	3	2
iPS DF 6.9 cell line	3	1
large intestine	3	3
liver	3	2
lung	3	3
muscle leg	3	3
muscle trunk	3	3
Neurosphere Cultured Cells Cortex Derived	2	4
Neurosphere Cultured Cells Ganglionic Eminence Derived	3	4
ovary	3	3
pancreas	3	3
Penis Foreskin Fibroblast Primary Cells	1	3
Penis Foreskin Keratinocyte Primary Cells	2	3
placenta	3	2
psoas muscle	3	3
sigmoid colon	3	3
small intestine	3	3
spinal cord	3	3
spleen	3	3

stomach	3	3
thymus	3	3
UCSF-4 embryonic stem cell line	2	2

Supplementary Table S6. **Enhancers = H3K27ac+H3K4me1-H3K4me3**

Name	Samples ids	TL num	BG num	BG std	Fisher Pvalue
A549	ENCFF697WAE, ENCFF276ZZL, ENCFF558FNN	915	376.66	20.59	3.6E-53
ascending aorta	ENCFF020COG, ENCFF838XQY, ENCFF865ATS	1544	749.42	24.28	3.9E-65
body of pancreas	ENCFF008FVK, ENCFF946VNI, ENCFF416CSY	1980	574.18	22.72	1.7E-186
breast epithelium	ENCFF190SES, ENCFF492VMK, ENCFF779MAU	278	115.84	10.56	9.0E-17
esophagus muscularis mucosa	ENCFF693CZV, ENCFF637MZI, ENCFF534AZK	1628	900.74	28.01	7.8E-50
esophagus squamous epithelium	ENCFF137CFG, ENCFF672JUL, ENCFF572LCU	1157	756.74	32.14	1.2E-20
gastrocnemius medialis	ENCFF094AJQ, ENCFF286NIM, ENCFF256NUL	2876	1527.04	36.88	1.4E-99
gastroesophageal sphincter	ENCFF601HAA, ENCFF626UWX, ENCFF060URO	138	62.38	8.46	7.7E-08
heart left ventricle	ENCFF946SRJ, ENCFF986INV, ENCFF391EFJ	3023	1174.48	35.20	4.3E-197
MM.1S	ENCFF363EKN, ENCFF538AHZ, ENCFF552LNJ	130	74.44	9.36	1.0E-04
neutrophil	ENCFF775EFB, ENCFF598KIH, ENCFF480KEJ	760	624.74	25.37	2.4E-4
Peyer's patch	ENCFF218SQX, ENCFF357CHH, ENCFF603AYP	59	17.02	4.58	1.4E-06
right atrium auricular region	ENCFF052BXS, ENCFF052PPU, ENCFF593MHZ	15	4.04	1.95	1.9E-2
right lobe of liver	ENCFF710URH, ENCFF629FSX, ENCFF636VXP	37	20.2	4.56	3.3E-2

sigmoid colon	ENCFF067IPT, ENCFF466OIS, ENCFF197CZF	109	28.72	5.92	1.7E-12
spleen	ENCFF640EAT, ENCFF837ZZH, ENCFF590BSY	485	275.78	16.16	1.7E-14
stomach	ENCFF888CZC, ENCFF735KDA, ENCFF612IVQ	2783	1011.6	31.12	7.9E-200
thoracic aorta	ENCFF003NBP, ENCFF080KQQ, ENCFF582FDJ	287	155.94	12.43	3.0E-10
thyroid gland	ENCFF302TBY, ENCFF710BOL, ENCFF767FGW	1871	748.42	24.86	2.8E-114
tibial nerve	ENCFF134SOM, ENCFF724DKP, ENCFF860VIA	1705	718.8	27.16	3.91E-95
transverse colon	ENCFF151PGH, ENCFF552DJF, ENCFF925YZC	188	68.66	9.31	3.1E-14

Supplementary Table S7. **Expression data source**

Tissue	Samples id	URL
adipose	SRR578647	`ftp://ftp.ncbi.nlm.nih.gov/sra/sra-instant/reads/ByRun/sra/SRR/SRR578/SRR578647`
adipose	SRR1045523	`ftp://ftp.ncbi.nlm.nih.gov/sra/sra-instant/reads/ByRun/sra/SRR/SRR104/SRR1045523`
adipose	SRR1045591	`ftp://ftp.ncbi.nlm.nih.gov/sra/sra-instant/reads/ByRun/sra/SRR/SRR104/SRR1045591`
adrenal gland	SRR578643	`ftp://ftp.ncbi.nlm.nih.gov/sra/sra-instant/reads/ByRun/sra/SRR/SRR578/SRR578643`
adrenal gland	SRR1045582	`ftp://ftp.ncbi.nlm.nih.gov/sra/sra-instant/reads/ByRun/sra/SRR/SRR104/SRR1045582`
adrenal gland	SRR644516	`ftp://ftp.ncbi.nlm.nih.gov/sra/sra-instant/reads/ByRun/sra/SRR/SRR644/SRR644516`
bladder	SRR651662	`ftp://ftp.ncbi.nlm.nih.gov/sra/sra-instant/reads/ByRun/sra/SRR/SRR651/SRR651662`
Brain Germinal Matrix	GSM751275	`ftp://ftp.ncbi.nlm.nih.gov/geo/samples/GSM751nnn/GSM751275/suppl/GSM751275%5FUCSF%2DUBC%2EBrain%5FGerminal%5FMatrix%2EmRNA%2DSeq%2EHuFGM02%2Ebed%2Egz`
Breast Luminal Epithelial Cells	GSM1127103	`ftp://ftp.ncbi.nlm.nih.gov/geo/samples/GSM1127nnn/GSM1127103/suppl/GSM1127103%5FUCSF%2DUBC%2EBreast%5FLuminal%5FEpithelial%5FCells%2EmRNA%2DSeq%2ERM084%2Ebed%2Egz`
Breast Luminal Epithelial Cells	GSM543029	`ftp://ftp.ncbi.nlm.nih.gov/geo/samples/GSM543nnn/GSM543029/suppl/GSM543029%5FUCSF%2DUBC%2EBreast%5FLuminal%5FEpithelial%5FCells%2EmRNA%2DSeq%2ERM035%2Ebed%2Egz`

Breast Luminal Epithelial Cells	GSM669620	ftp://ftp.ncbi.nlm.nih.gov/geo/samples/GSM669nnn/GSM669620/suppl/GSM669620%5FUCSF%2DUBC%2EBreast%5FLuminal%5FEpithelial%5FCells%2EmRNA%2DSeq%2ERM080%2Ebed%2Egz
Breast Myoepithelial Cells	GSM1127093	ftp://ftp.ncbi.nlm.nih.gov/geo/samples/GSM1127nnn/GSM1127093/suppl/GSM1127093%5FUCSF%2DUBC%2EBreast%5FMyoepithelial%5FCells%2EmRNA%2DSeq%2ERM084%2Ebed%2Egz
Breast Myoepithelial Cells	GSM543031	ftp://ftp.ncbi.nlm.nih.gov/geo/samples/GSM543nnn/GSM543031/suppl/GSM543031%5FUCSF%2DUBC%2EBreast%5FMyoepithelial%5FCells%2EmRNA%2DSeq%2ERM035%2Ebed%2Egz
Breast Myoepithelial Cells	GSM669621	ftp://ftp.ncbi.nlm.nih.gov/geo/samples/GSM669nnn/GSM669621/suppl/GSM669621%5FUCSF%2DUBC%2EBreast%5FMyoepithelial%5FCells%2EmRNA%2DSeq%2ERM080%2Ebed%2Egz
CD14 primary cells	SRR980470	ftp://ftp.ncbi.nlm.nih.gov/sra/sra-instant/reads/ByRun/sra/SRR/SRR980/SRR980470
CD3 primary cells	SRR980468	ftp://ftp.ncbi.nlm.nih.gov/sra/sra-instant/reads/ByRun/sra/SRR/SRR980/SRR980468
CD3 primary cells	SRR980469	ftp://ftp.ncbi.nlm.nih.gov/sra/sra-instant/reads/ByRun/sra/SRR/SRR980/SRR980469
CD56 primary cells	SRR980472	ftp://ftp.ncbi.nlm.nih.gov/sra/sra-instant/reads/ByRun/sra/SRR/SRR980/SRR980472
esophagus	SRR596102	ftp://ftp.ncbi.nlm.nih.gov/sra/sra-instant/reads/ByRun/sra/SRR/SRR596/SRR596102
esophagus	SRR596103	ftp://ftp.ncbi.nlm.nih.gov/sra/sra-instant/reads/ByRun/sra/SRR/SRR596/SRR596103
esophagus	SRR1045586	ftp://ftp.ncbi.nlm.nih.gov/sra/sra-instant/reads/ByRun/sra/SRR/SRR104/SRR1045586
gastric	SRR577583	ftp://ftp.ncbi.nlm.nih.gov/sra/sra-instant/reads/ByRun/sra/SRR/SRR577/SRR577583
gastric	SRR1045527	ftp://ftp.ncbi.nlm.nih.gov/sra/sra-instant/reads/ByRun/sra/SRR/SRR104/SRR1045527
gastric	SRR1045595	ftp://ftp.ncbi.nlm.nih.gov/sra/sra-instant/reads/ByRun/sra/SRR/SRR104/SRR1045595
H1 +BMP4 cell line	SRR179591	ftp://ftp.ncbi.nlm.nih.gov/sra/sra-instant/reads/ByRun/sra/SRR/SRR179/SRR179591
H1 +BMP4 cell line	SRR179592	ftp://ftp.ncbi.nlm.nih.gov/sra/sra-instant/reads/ByRun/sra/SRR/SRR179/SRR179592
H1 BMP4 derived mesendoderm cultured cells	SRR488136	ftp://ftp.ncbi.nlm.nih.gov/sra/sra-instant/reads/ByRun/sra/SRR/SRR488/SRR488136
H1 BMP4 derived mesendoderm cultured cells	SRR488137	ftp://ftp.ncbi.nlm.nih.gov/sra/sra-instant/reads/ByRun/sra/SRR/SRR488/SRR488137
H1 cell line	SRR020289	ftp://ftp.ncbi.nlm.nih.gov/sra/sra-instant/reads/ByRun/sra/SRR/SRR020/SRR020289

H1 cell line	SRR031628	https://trace.ncbi.nlm.nih.gov/Traces/sra/sra.cgi?run=SRR031628
H1 cell line	SRR488684	ftp://ftp.ncbi.nlm.nih.gov/sra/sra-instant/reads/ByRun/sra/SRR/SRR488/SRR488684
H1 derived mesenchymal stem cells	SRR486239	ftp://ftp.ncbi.nlm.nih.gov/sra/sra-instant/reads/ByRun/sra/SRR/SRR486/SRR486239
H1 derived mesenchymal stem cells	SRR486240	ftp://ftp.ncbi.nlm.nih.gov/sra/sra-instant/reads/ByRun/sra/SRR/SRR486/SRR486240
H1 derived neuronal progenitor cultured cells	SRR179593	ftp://ftp.ncbi.nlm.nih.gov/sra/sra-instant/reads/ByRun/sra/SRR/SRR179/SRR179593
H1 derived neuronal progenitor cultured cells	SRR179594	ftp://ftp.ncbi.nlm.nih.gov/sra/sra-instant/reads/ByRun/sra/SRR/SRR179/SRR179594
H1 derived neuronal progenitor cultured cells	SRR486241	ftp://ftp.ncbi.nlm.nih.gov/sra/sra-instant/reads/ByRun/sra/SRR/SRR486/SRR486241
H9 cell line	SRR304995	ftp://ftp.ncbi.nlm.nih.gov/sra/sra-instant/reads/ByRun/sra/SRR/SRR304/SRR304995
heart aorta	SRR596098	ftp://ftp.ncbi.nlm.nih.gov/sra/sra-instant/reads/ByRun/sra/SRR/SRR596/SRR596098
heart aorta	SRR596099	ftp://ftp.ncbi.nlm.nih.gov/sra/sra-instant/reads/ByRun/sra/SRR/SRR596/SRR596099
heart aorta	SRR1054856	ftp://ftp.ncbi.nlm.nih.gov/sra/sra-instant/reads/ByRun/sra/SRR/SRR105/SRR1054856
heart left ventricle	SRR578627	ftp://ftp.ncbi.nlm.nih.gov/sra/sra-instant/reads/ByRun/sra/SRR/SRR578/SRR578627
heart left ventricle	SRR578628	ftp://ftp.ncbi.nlm.nih.gov/sra/sra-instant/reads/ByRun/sra/SRR/SRR578/SRR578628
heart left ventricle	SRR577587	ftp://ftp.ncbi.nlm.nih.gov/sra/sra-instant/reads/ByRun/sra/SRR/SRR577/SRR577587
heart right atrium	SRR578651	ftp://ftp.ncbi.nlm.nih.gov/sra/sra-instant/reads/ByRun/sra/SRR/SRR578/SRR578651
heart right atrium	SRR578652	ftp://ftp.ncbi.nlm.nih.gov/sra/sra-instant/reads/ByRun/sra/SRR/SRR578/SRR578652
heart right atrium	SRR578653	ftp://ftp.ncbi.nlm.nih.gov/sra/sra-instant/reads/ByRun/sra/SRR/SRR578/SRR578653
heart right ventricle	SRR577591	ftp://ftp.ncbi.nlm.nih.gov/sra/sra-instant/reads/ByRun/sra/SRR/SRR577/SRR577591
heart right ventricle	SRR577592	ftp://ftp.ncbi.nlm.nih.gov/sra/sra-instant/reads/ByRun/sra/SRR/SRR577/SRR577592
heart right ventricle	SRR1045574	ftp://ftp.ncbi.nlm.nih.gov/sra/sra-instant/reads/ByRun/sra/SRR/SRR104/SRR1045574
hESC-derived CD184+ endoderm cultured cells	SRR1067051	ftp://ftp.ncbi.nlm.nih.gov/sra/sra-instant/reads/ByRun/sra/SRR/SRR106/SRR1067051

hESC-derived CD184+ endoderm cultured cells	SRR1067055	ftp://ftp.ncbi.nlm.nih.gov/sra/sra-instant/reads/ByRun/sra/SRR/SRR106/SRR1067055
hESC-derived CD184+ endoderm cultured cells	SRR1067049	ftp://ftp.ncbi.nlm.nih.gov/sra/sra-instant/reads/ByRun/sra/SRR/SRR106/SRR1067049
hESC-derived CD56+ ectoderm cultured cells	SRR1097443	ftp://ftp.ncbi.nlm.nih.gov/sra/sra-instant/reads/ByRun/sra/SRR/SRR109/SRR1097443
hESC-derived CD56+ ectoderm cultured cells	SRR1097446	ftp://ftp.ncbi.nlm.nih.gov/sra/sra-instant/reads/ByRun/sra/SRR/SRR109/SRR1097446
hESC-derived CD56+ ectoderm cultured cells	SRR1097444	ftp://ftp.ncbi.nlm.nih.gov/sra/sra-instant/reads/ByRun/sra/SRR/SRR109/SRR1097444
hESC-derived CD56+ mesoderm cultured cells	SRR1067489	ftp://ftp.ncbi.nlm.nih.gov/sra/sra-instant/reads/ByRun/sra/SRR/SRR106/SRR1067489
hESC-derived CD56+ mesoderm cultured cells	SRR1067490	ftp://ftp.ncbi.nlm.nih.gov/sra/sra-instant/reads/ByRun/sra/SRR/SRR106/SRR1067490
hESC-derived CD56+ mesoderm cultured cells	SRR1067491	ftp://ftp.ncbi.nlm.nih.gov/sra/sra-instant/reads/ByRun/sra/SRR/SRR106/SRR1067491
HUES64 cell line	SRR1107846	ftp://ftp.ncbi.nlm.nih.gov/sra/sra-instant/reads/ByRun/sra/SRR/SRR110/SRR1107846
HUES64 cell line	SRR1107848	ftp://ftp.ncbi.nlm.nih.gov/sra/sra-instant/reads/ByRun/sra/SRR/SRR110/SRR1107848
HUES64 cell line	SRR1067487	ftp://ftp.ncbi.nlm.nih.gov/sra/sra-instant/reads/ByRun/sra/SRR/SRR106/SRR1067487
IMR90 cell line	SRR020295	ftp://ftp.ncbi.nlm.nih.gov/sra/sra-instant/reads/ByRun/sra/SRR/SRR020/SRR020295
IMR90 cell line	SRR020293	ftp://ftp.ncbi.nlm.nih.gov/sra/sra-instant/reads/ByRun/sra/SRR/SRR020/SRR020293
IMR90 cell line	SRR020294	ftp://ftp.ncbi.nlm.nih.gov/sra/sra-instant/reads/ByRun/sra/SRR/SRR020/SRR020294
iPS DF 19.11 cell line	SRR179588	ftp://ftp.ncbi.nlm.nih.gov/sra/sra-instant/reads/ByRun/sra/SRR/SRR179/SRR179588
iPS DF 19.11 cell line	SRR179589	ftp://ftp.ncbi.nlm.nih.gov/sra/sra-instant/reads/ByRun/sra/SRR/SRR179/SRR179589
iPS DF 6.9 cell line	SRR179590	ftp://ftp.ncbi.nlm.nih.gov/sra/sra-instant/reads/ByRun/sra/SRR/SRR179/SRR179590

large intestine	SRR643761	ftp://ftp.ncbi.nlm.nih.gov/sra/sra-instant/reads/ByRun/sra/SRR/SRR643/SRR643761
large intestine	SRR643741	ftp://ftp.ncbi.nlm.nih.gov/sra/sra-instant/reads/ByRun/sra/SRR/SRR643/SRR643741
large intestine	SRR643755	ftp://ftp.ncbi.nlm.nih.gov/sra/sra-instant/reads/ByRun/sra/SRR/SRR643/SRR643755
liver	SRR651663	ftp://ftp.ncbi.nlm.nih.gov/sra/sra-instant/reads/ByRun/sra/SRR/SRR651/SRR651663
liver	SRR651664	ftp://ftp.ncbi.nlm.nih.gov/sra/sra-instant/reads/ByRun/sra/SRR/SRR651/SRR651664
lung	SRR577579	ftp://ftp.ncbi.nlm.nih.gov/sra/sra-instant/reads/ByRun/sra/SRR/SRR577/SRR577579
lung	SRR577580	ftp://ftp.ncbi.nlm.nih.gov/sra/sra-instant/reads/ByRun/sra/SRR/SRR577/SRR577580
lung	SRR1045530	ftp://ftp.ncbi.nlm.nih.gov/sra/sra-instant/reads/ByRun/sra/SRR/SRR104/SRR1045530
muscle leg	SRR786797	ftp://ftp.ncbi.nlm.nih.gov/sra/sra-instant/reads/ByRun/sra/SRR/SRR786/SRR786797
muscle leg	SRR786777	ftp://ftp.ncbi.nlm.nih.gov/sra/sra-instant/reads/ByRun/sra/SRR/SRR786/SRR786777
muscle leg	SRR980475	ftp://ftp.ncbi.nlm.nih.gov/sra/sra-instant/reads/ByRun/sra/SRR/SRR980/SRR980475
muscle trunk	SRR980485	ftp://ftp.ncbi.nlm.nih.gov/sra/sra-instant/reads/ByRun/sra/SRR/SRR980/SRR980485
muscle trunk	SRR980486	ftp://ftp.ncbi.nlm.nih.gov/sra/sra-instant/reads/ByRun/sra/SRR/SRR980/SRR980486
muscle trunk	SRR786757	ftp://ftp.ncbi.nlm.nih.gov/sra/sra-instant/reads/ByRun/sra/SRR/SRR786/SRR786757
Neurosphere Cultured Cells Cortex Derived	GSM751270	ftp://ftp.ncbi.nlm.nih.gov/geo/samples/GSM751nnn/GSM751270/suppl/GSM751270%5FUCSF%2DUBC%2ENeurosphere%5FCultured%5FCells%5FCortex%5FDerived%2EmRNA%2DSeq%2EHuFNSC01%2Ebed%2Egz
Neurosphere Cultured Cells Cortex Derived	GSM751272	ftp://ftp.ncbi.nlm.nih.gov/geo/samples/GSM751nnn/GSM751272/suppl/GSM751272%5FUCSF%2DUBC%2ENeurosphere%5FCultured%5FCells%5FCortex%5FDerived%2EmRNA%2DSeq%2EHuFNSC02%2Ebed%2Egz
Neurosphere Cultured Cells Cortex Derived	GSM751279	ftp://ftp.ncbi.nlm.nih.gov/geo/samples/GSM751nnn/GSM751279/suppl/GSM751279%5FUCSF%2DUBC%2ENeurosphere%5FCultured%5FCells%5FCortex%5FDerived%2EmRNA%2DSeq%2EHuFNSC03%2Ebed%2Egz
Neurosphere Cultured Cells Cortex Derived	GSM958176	ftp://ftp.ncbi.nlm.nih.gov/geo/samples/GSM958nnn/GSM958176/suppl/GSM958176%5FUCSF%2DUBC%2ENeurosphere%5FCultured%5FCells%5FCortex%5FDerived%2EmRNA%2DSeq%2EHuFNSC04%2Ebed%2Egz

Neurosphere Cultured Cells Ganglionic Eminence Derived	GSM751271	ftp://ftp.ncbi.nlm.nih.gov/geo/samples/GSM751nnn/GSM751271/suppl/GSM751271%5FUCSF%2DUBC%2ENeurosphere%5FCultured%5FCells%5FGanglionic%5FEminence%5FDerived%2EmRNA%2DSeq%2EHuFNSC01%2Ebed%2Egz
Neurosphere Cultured Cells Ganglionic Eminence Derived	GSM751273	ftp://ftp.ncbi.nlm.nih.gov/geo/samples/GSM751nnn/GSM751273/suppl/GSM751273%5FUCSF%2DUBC%2ENeurosphere%5FCultured%5FCells%5FGanglionic%5FEminence%5FDerived%2EmRNA%2DSeq%2EHuFNSC02%2Ebed%2Egz
Neurosphere Cultured Cells Ganglionic Eminence Derived	GSM958172	ftp://ftp.ncbi.nlm.nih.gov/geo/samples/GSM958nnn/GSM958172/suppl/GSM958172%5FUCSF%2DUBC%2ENeurosphere%5FCultured%5FCells%5FGanglionic%5FEminence%5FDerived%2EmRNA%2DSeq%2EHuFNSC04%2Ebed%2Egz
Neurosphere Cultured Cells Ganglionic Eminence Derived	GSM958173	ftp://ftp.ncbi.nlm.nih.gov/geo/samples/GSM958nnn/GSM958173/suppl/GSM958173%5FUCSF%2DUBC%2ENeurosphere%5FCultured%5FCells%5FGanglionic%5FEminence%5FDerived%2EmRNA%2DSeq%2EHuFNSC03%2Ebed%2Egz
ovary	SRR578639	ftp://ftp.ncbi.nlm.nih.gov/sra/sra-instant/reads/ByRun/sra/SRR/SRR578/SRR578639
ovary	SRR578640	ftp://ftp.ncbi.nlm.nih.gov/sra/sra-instant/reads/ByRun/sra/SRR/SRR578/SRR578640
ovary	SRR786786	ftp://ftp.ncbi.nlm.nih.gov/sra/sra-instant/reads/ByRun/sra/SRR/SRR786/SRR786786
pancreas	SRR596106	ftp://ftp.ncbi.nlm.nih.gov/sra/sra-instant/reads/ByRun/sra/SRR/SRR596/SRR596106
pancreas	SRR596107	ftp://ftp.ncbi.nlm.nih.gov/sra/sra-instant/reads/ByRun/sra/SRR/SRR596/SRR596107
pancreas	SRR1045598	ftp://ftp.ncbi.nlm.nih.gov/sra/sra-instant/reads/ByRun/sra/SRR/SRR104/SRR1045598
Penis Foreskin Fibroblast Primary Cells	GSM751277	ftp://ftp.ncbi.nlm.nih.gov/geo/samples/GSM751nnn/GSM751277/suppl/GSM751277%5FUCSF%2DUBC%2EPenis%5FForeskin%5FFibroblast%5FPrimary%5FCells%2EmRNA%2DSeq%2Eskin01%2Ebed%2Egz
Penis Foreskin Fibroblast Primary Cells	GSM941744	ftp://ftp.ncbi.nlm.nih.gov/geo/samples/GSM941nnn/GSM941744/suppl/GSM941744%5FUCSF%2DUBC%2EPenis%5FForeskin%5FFibroblast%5FPrimary%5FCells%2EmRNA%2DSeq%2Eskin02%2Ebed%2Egz
Penis Foreskin Fibroblast Primary Cells	GSM958178	ftp://ftp.ncbi.nlm.nih.gov/geo/samples/GSM958nnn/GSM958178/suppl/GSM958178%5FUCSF%2DUBC%2EPenis%5FForeskin%5FFibroblast%5FPrimary%5FCells%2EmRNA%2DSeq%2Eskin03%2Ebed%2Egz

Penis Foreskin Keratinocyte Primary Cells	GSM958177	ftp://ftp.ncbi.nlm.nih.gov/geo/samples/GSM958nnn/GSM958177/suppl/GSM958177%5FUCSF%2DUBC%2EPenis%5FForeskin%5FKeratinocyte%5FPrimary%5FCells%2EmRNA%2DSeq%2Eskin03%2Ebed%2Egz
Penis Foreskin Keratinocyte Primary Cells	GSM751278	ftp://ftp.ncbi.nlm.nih.gov/geo/samples/GSM751nnn/GSM751278/suppl/GSM751278%5FUCSF%2DUBC%2EPenis%5FForeskin%5FKeratinocyte%5FPrimary%5FCells%2EmRNA%2DSeq%2Eskin01%2Ebed%2Egz
Penis Foreskin Keratinocyte Primary Cells	GSM941745	ftp://ftp.ncbi.nlm.nih.gov/geo/samples/GSM941nnn/GSM941745/suppl/GSM941745%5FUCSF%2DUBC%2EPenis%5FForeskin%5FKeratinocyte%5FPrimary%5FCells%2EmRNA%2DSeq%2Eskin02%2Ebed%2Egz
placenta	SRR980480	ftp://ftp.ncbi.nlm.nih.gov/sra/sra-instant/reads/ByRun/sra/SRR/SRR980/SRR980480
placenta	SRR980481	ftp://ftp.ncbi.nlm.nih.gov/sra/sra-instant/reads/ByRun/sra/SRR/SRR980/SRR980481
psoas muscle	SRR651699	ftp://ftp.ncbi.nlm.nih.gov/sra/sra-instant/reads/ByRun/sra/SRR/SRR651/SRR651699
psoas muscle	SRR1045571	ftp://ftp.ncbi.nlm.nih.gov/sra/sra-instant/reads/ByRun/sra/SRR/SRR104/SRR1045571
psoas muscle	SRR1045603	ftp://ftp.ncbi.nlm.nih.gov/sra/sra-instant/reads/ByRun/sra/SRR/SRR104/SRR1045603
sigmoid colon	SRR578635	ftp://ftp.ncbi.nlm.nih.gov/sra/sra-instant/reads/ByRun/sra/SRR/SRR578/SRR578635
sigmoid colon	SRR577595	ftp://ftp.ncbi.nlm.nih.gov/sra/sra-instant/reads/ByRun/sra/SRR/SRR577/SRR577595
sigmoid colon	SRR1045610	ftp://ftp.ncbi.nlm.nih.gov/sra/sra-instant/reads/ByRun/sra/SRR/SRR104/SRR1045610
small intestine	SRR578631	ftp://ftp.ncbi.nlm.nih.gov/sra/sra-instant/reads/ByRun/sra/SRR/SRR578/SRR578631
small intestine	SRR1045607	ftp://ftp.ncbi.nlm.nih.gov/sra/sra-instant/reads/ByRun/sra/SRR/SRR104/SRR1045607
small intestine	SRR1045619	ftp://ftp.ncbi.nlm.nih.gov/sra/sra-instant/reads/ByRun/sra/SRR/SRR104/SRR1045619
spinal cord	SRR786783	ftp://ftp.ncbi.nlm.nih.gov/sra/sra-instant/reads/ByRun/sra/SRR/SRR786/SRR786783
spinal cord	SRR980477	ftp://ftp.ncbi.nlm.nih.gov/sra/sra-instant/reads/ByRun/sra/SRR/SRR980/SRR980477
spinal cord	SRR786804	ftp://ftp.ncbi.nlm.nih.gov/sra/sra-instant/reads/ByRun/sra/SRR/SRR786/SRR786804
spleen	SRR577599	ftp://ftp.ncbi.nlm.nih.gov/sra/sra-instant/reads/ByRun/sra/SRR/SRR577/SRR577599
spleen	SRR1045579	ftp://ftp.ncbi.nlm.nih.gov/sra/sra-instant/reads/ByRun/sra/SRR/SRR104/SRR1045579
spleen	SRR1045615	ftp://ftp.ncbi.nlm.nih.gov/sra/sra-instant/reads/ByRun/sra/SRR/SRR104/SRR1045615
stomach	SRR980462	ftp://ftp.ncbi.nlm.nih.gov/sra/sra-instant/reads/ByRun/sra/SRR/SRR980/SRR980462

stomach	SRR980459	ftp://ftp.ncbi.nlm.nih.gov/sra/sra-instant/reads/ByRun/sra/SRR/SRR980/SRR980459
stomach	SRR980451	ftp://ftp.ncbi.nlm.nih.gov/sra/sra-instant/reads/ByRun/sra/SRR/SRR980/SRR980451
thymus	SRR651673	ftp://ftp.ncbi.nlm.nih.gov/sra/sra-instant/reads/ByRun/sra/SRR/SRR651/SRR651673
thymus	SRR980452	ftp://ftp.ncbi.nlm.nih.gov/sra/sra-instant/reads/ByRun/sra/SRR/SRR980/SRR980452
thymus	SRR980473	ftp://ftp.ncbi.nlm.nih.gov/sra/sra-instant/reads/ByRun/sra/SRR/SRR980/SRR980473
UCSF-4 embryonic stem cell line	GSM958175	ftp://ftp.ncbi.nlm.nih.gov/geo/samples/GSM958nnn/GSM958175/suppl/GSM958175%5FUCSF%2DUBC%2EUCSF%2D4star%2EmRNA%2DSeq%2EA11832%2Ebed%2Egz
UCSF-4 embryonic stem cell line	GSM958179	ftp://ftp.ncbi.nlm.nih.gov/geo/samples/GSM958nnn/GSM958179/suppl/GSM958179%5FUCSF%2DUBC%2EUCSF%2D4star%2EmRNA%2DSeq%2EA11831%2Ebed%2Egz

Supplementary Table S8. Methylation data source

Tissue	Samples id	URL
adipose	SRR577617	ftp://ftp.ncbi.nlm.nih.gov/sra/sra-instant/reads/ByRun/sra/SRR/SRR577/SRR577617
adipose	SRR1045747	ftp://ftp.ncbi.nlm.nih.gov/sra/sra-instant/reads/ByRun/sra/SRR/SRR104/SRR1045747
adipose	SRR1045745	ftp://ftp.ncbi.nlm.nih.gov/sra/sra-instant/reads/ByRun/sra/SRR/SRR104/SRR1045745
adrenal gland	SRR577613	ftp://ftp.ncbi.nlm.nih.gov/sra/sra-instant/reads/ByRun/sra/SRR/SRR577/SRR577613
adrenal gland	SRR1045733	ftp://ftp.ncbi.nlm.nih.gov/sra/sra-instant/reads/ByRun/sra/SRR/SRR104/SRR1045733
adrenal gland	SRR1045734	ftp://ftp.ncbi.nlm.nih.gov/sra/sra-instant/reads/ByRun/sra/SRR/SRR104/SRR1045734
bladder	SRR641595	ftp://ftp.ncbi.nlm.nih.gov/sra/sra-instant/reads/ByRun/sra/SRR/SRR641/SRR641595
bladder	SRR641596	ftp://ftp.ncbi.nlm.nih.gov/sra/sra-instant/reads/ByRun/sra/SRR/SRR641/SRR641596
bladder	SRR1045744	ftp://ftp.ncbi.nlm.nih.gov/sra/sra-instant/reads/ByRun/sra/SRR/SRR104/SRR1045744
Brain Germinal Matrix	GSM941747	ftp://ftp.ncbi.nlm.nih.gov/geo/samples/GSM941nnn/GSM941747/suppl/GSM941747%5FUCSF%2DUBC%2EBrain%5FGerminal%5FMatrix%2EBisulfite%2DSeq%2EHuFGM02%2Ewig%2Egz
Breast Luminal Epithelial Cells	GSM1127125	ftp://ftp.ncbi.nlm.nih.gov/geo/samples/GSM1127nnn/GSM1127125/suppl/GSM1127125%5FUCSF%2DUBC%2EBreast%5FLuminal%5FEpithelial%5FCells%2EBisulfite%2DSeq%2ERM066%2Ewig%2Egz

Breast Myoepithelial Cells	GSM1127054	ftp://ftp.ncbi.nlm.nih.gov/geo/samples/GSM1127nnn/GSM1127054/suppl/GSM1127054%5FUCSF%2DUBC%2EBreast%5FMyoepithelial%5FCells%2EBisulfite%2DSeq%2ERM066%2Ewig%2Egz
Breast Myoepithelial Cells	GSM1127057	ftp://ftp.ncbi.nlm.nih.gov/geo/samples/GSM1127nnn/GSM1127057/suppl/GSM1127057%5FUCSF%2DUBC%2EBreast%5FMyoepithelial%5FCells%2EBisulfite%2DSeq%2ERM066%2Ewig%2Egz
Breast Myoepithelial Cells	GSM1127059	ftp://ftp.ncbi.nlm.nih.gov/geo/samples/GSM1127nnn/GSM1127059/suppl/GSM1127059%5FUCSF%2DUBC%2EBreast%5FMyoepithelial%5FCells%2EBisulfite%2DSeq%2ERM066%2Ewig%2Egz
CD14 primary cells	SRR1104848	ftp://ftp.ncbi.nlm.nih.gov/sra/sra-instant/reads/ByRun/sra/SRR/SRR110/SRR1104848
CD14 primary cells	SRR1104855	ftp://ftp.ncbi.nlm.nih.gov/sra/sra-instant/reads/ByRun/sra/SRR/SRR110/SRR1104855
CD14 primary cells	SRR1104856	ftp://ftp.ncbi.nlm.nih.gov/sra/sra-instant/reads/ByRun/sra/SRR/SRR110/SRR1104856
CD3 primary cells	SRR1104838	ftp://ftp.ncbi.nlm.nih.gov/sra/sra-instant/reads/ByRun/sra/SRR/SRR110/SRR1104838
CD3 primary cells	SRR1104839	ftp://ftp.ncbi.nlm.nih.gov/sra/sra-instant/reads/ByRun/sra/SRR/SRR110/SRR1104839
CD3 primary cells	SRR1104841	ftp://ftp.ncbi.nlm.nih.gov/sra/sra-instant/reads/ByRun/sra/SRR/SRR110/SRR1104841
CD56 primary cells	SRR1104851	ftp://ftp.ncbi.nlm.nih.gov/sra/sra-instant/reads/ByRun/sra/SRR/SRR110/SRR1104851
CD56 primary cells	SRR1104862	ftp://ftp.ncbi.nlm.nih.gov/sra/sra-instant/reads/ByRun/sra/SRR/SRR110/SRR1104862
CD56 primary cells	SRR1104863	ftp://ftp.ncbi.nlm.nih.gov/sra/sra-instant/reads/ByRun/sra/SRR/SRR110/SRR1104863
esophagus	SRR536241	ftp://ftp.ncbi.nlm.nih.gov/sra/sra-instant/reads/ByRun/sra/SRR/SRR536/SRR536241
esophagus	SRR547638	ftp://ftp.ncbi.nlm.nih.gov/sra/sra-instant/reads/ByRun/sra/SRR/SRR547/SRR547638
esophagus	SRR1045682	ftp://ftp.ncbi.nlm.nih.gov/sra/sra-instant/reads/ByRun/sra/SRR/SRR104/SRR1045682
gastric	SRR577621	ftp://ftp.ncbi.nlm.nih.gov/sra/sra-instant/reads/ByRun/sra/SRR/SRR577/SRR577621
gastric	SRR1045750	ftp://ftp.ncbi.nlm.nih.gov/sra/sra-instant/reads/ByRun/sra/SRR/SRR104/SRR1045750
gastric	SRR1045749	ftp://ftp.ncbi.nlm.nih.gov/sra/sra-instant/reads/ByRun/sra/SRR/SRR104/SRR1045749
H1 +BMP4 cell line	SRR067722	ftp://ftp.ncbi.nlm.nih.gov/sra/sra-instant/reads/ByRun/sra/SRR/SRR067/SRR067722
H1 +BMP4 cell line	SRR067729	ftp://ftp.ncbi.nlm.nih.gov/sra/sra-instant/reads/ByRun/sra/SRR/SRR067/SRR067729
H1 +BMP4 cell line	SRR067730	ftp://ftp.ncbi.nlm.nih.gov/sra/sra-instant/reads/ByRun/sra/SRR/SRR067/SRR067730

H1 BMP4 derived mesendoderm cultured cells	SRR364089	ftp://ftp.ncbi.nlm.nih.gov/sra/sra-instant/reads/ByRun/sra/SRR/SRR364/SRR364089
H1 BMP4 derived mesendoderm cultured cells	SRR364317	ftp://ftp.ncbi.nlm.nih.gov/sra/sra-instant/reads/ByRun/sra/SRR/SRR364/SRR364317
H1 BMP4 derived mesendoderm cultured cells	SRR364090	ftp://ftp.ncbi.nlm.nih.gov/sra/sra-instant/reads/ByRun/sra/SRR/SRR364/SRR364090
H1 cell line	SRR019072	ftp://ftp.ncbi.nlm.nih.gov/sra/sra-instant/reads/ByRun/sra/SRR/SRR019/SRR019072
H1 cell line	SRR018975	ftp://ftp.ncbi.nlm.nih.gov/sra/sra-instant/reads/ByRun/sra/SRR/SRR018/SRR018975
H1 cell line	SRR020282	ftp://ftp.ncbi.nlm.nih.gov/sra/sra-instant/reads/ByRun/sra/SRR/SRR020/SRR020282
H1 derived mesenchymal stem cells	SRR400557	ftp://ftp.ncbi.nlm.nih.gov/sra/sra-instant/reads/ByRun/sra/SRR/SRR400/SRR400557
H1 derived mesenchymal stem cells	SRR400558	ftp://ftp.ncbi.nlm.nih.gov/sra/sra-instant/reads/ByRun/sra/SRR/SRR400/SRR400558
H1 derived mesenchymal stem cells	SRR400566	ftp://ftp.ncbi.nlm.nih.gov/sra/sra-instant/reads/ByRun/sra/SRR/SRR400/SRR400566
H1 derived neuronal progenitor cultured cells	SRR101296	ftp://ftp.ncbi.nlm.nih.gov/sra/sra-instant/reads/ByRun/sra/SRR/SRR101/SRR101296
H1 derived neuronal progenitor cultured cells	SRR101297	ftp://ftp.ncbi.nlm.nih.gov/sra/sra-instant/reads/ByRun/sra/SRR/SRR101/SRR101297
H1 derived neuronal progenitor cultured cells	SRR101298	ftp://ftp.ncbi.nlm.nih.gov/sra/sra-instant/reads/ByRun/sra/SRR/SRR101/SRR101298
H9 cell line	SRR179602	ftp://ftp.ncbi.nlm.nih.gov/sra/sra-instant/reads/ByRun/sra/SRR/SRR179/SRR179602
H9 cell line	SRR179638	ftp://ftp.ncbi.nlm.nih.gov/sra/sra-instant/reads/ByRun/sra/SRR/SRR179/SRR179638
H9 cell line	SRR179668	ftp://ftp.ncbi.nlm.nih.gov/sra/sra-instant/reads/ByRun/sra/SRR/SRR179/SRR179668
heart aorta	SRR536239	ftp://ftp.ncbi.nlm.nih.gov/sra/sra-instant/reads/ByRun/sra/SRR/SRR536/SRR536239
heart aorta	SRR1045736	ftp://ftp.ncbi.nlm.nih.gov/sra/sra-instant/reads/ByRun/sra/SRR/SRR104/SRR1045736
heart aorta	SRR1045738	ftp://ftp.ncbi.nlm.nih.gov/sra/sra-instant/reads/ByRun/sra/SRR/SRR104/SRR1045738
heart left ventricle	SRR536242	ftp://ftp.ncbi.nlm.nih.gov/sra/sra-instant/reads/ByRun/sra/SRR/SRR536/SRR536242

heart left ventricle	SRR578655	ftp://ftp.ncbi.nlm.nih.gov/sra/sra-instant/reads/ByRun/sra/SRR/SRR578/SRR578655
heart left ventricle	SRR1045642	ftp://ftp.ncbi.nlm.nih.gov/sra/sra-instant/reads/ByRun/sra/SRR/SRR104/SRR1045642
heart right atrium	SRR577629	ftp://ftp.ncbi.nlm.nih.gov/sra/sra-instant/reads/ByRun/sra/SRR/SRR577/SRR577629
heart right atrium	SRR1045753	ftp://ftp.ncbi.nlm.nih.gov/sra/sra-instant/reads/ByRun/sra/SRR/SRR104/SRR1045753
heart right atrium	SRR1045752	ftp://ftp.ncbi.nlm.nih.gov/sra/sra-instant/reads/ByRun/sra/SRR/SRR104/SRR1045752
heart right ventricle	SRR577634	ftp://ftp.ncbi.nlm.nih.gov/sra/sra-instant/reads/ByRun/sra/SRR/SRR577/SRR577634
heart right ventricle	SRR651709	ftp://ftp.ncbi.nlm.nih.gov/sra/sra-instant/reads/ByRun/sra/SRR/SRR651/SRR651709
heart right ventricle	SRR1045756	ftp://ftp.ncbi.nlm.nih.gov/sra/sra-instant/reads/ByRun/sra/SRR/SRR104/SRR1045756
hESC-derived CD184+ endoderm cultured cells	SRR1143696	ftp://ftp.ncbi.nlm.nih.gov/sra/sra-instant/reads/ByRun/sra/SRR/SRR114/SRR1143696
hESC-derived CD184+ endoderm cultured cells	SRR1143697	ftp://ftp.ncbi.nlm.nih.gov/sra/sra-instant/reads/ByRun/sra/SRR/SRR114/SRR1143697
hESC-derived CD184+ endoderm cultured cells	SRR1067575	ftp://ftp.ncbi.nlm.nih.gov/sra/sra-instant/reads/ByRun/sra/SRR/SRR106/SRR1067575
hESC-derived CD56+ ectoderm cultured cells	SRR1067550	ftp://ftp.ncbi.nlm.nih.gov/sra/sra-instant/reads/ByRun/sra/SRR/SRR106/SRR1067550
hESC-derived CD56+ ectoderm cultured cells	SRR1067553	ftp://ftp.ncbi.nlm.nih.gov/sra/sra-instant/reads/ByRun/sra/SRR/SRR106/SRR1067553
hESC-derived CD56+ ectoderm cultured cells	SRR1067572	ftp://ftp.ncbi.nlm.nih.gov/sra/sra-instant/reads/ByRun/sra/SRR/SRR106/SRR1067572
hESC-derived CD56+ mesoderm cultured cells	SRR1067566	ftp://ftp.ncbi.nlm.nih.gov/sra/sra-instant/reads/ByRun/sra/SRR/SRR106/SRR1067566
hESC-derived CD56+ mesoderm cultured cells	SRR1067568	ftp://ftp.ncbi.nlm.nih.gov/sra/sra-instant/reads/ByRun/sra/SRR/SRR106/SRR1067568

hESC-derived CD56+ mesoderm cultured cells	SRR1067558	ftp://ftp.ncbi.nlm.nih.gov/sra/sra-instant/reads/ByRun/sra/SRR/SRR106/SRR1067558
HUES64 cell line	SRR1067557	ftp://ftp.ncbi.nlm.nih.gov/sra/sra-instant/reads/ByRun/sra/SRR/SRR106/SRR1067557
HUES64 cell line	SRR1067560	ftp://ftp.ncbi.nlm.nih.gov/sra/sra-instant/reads/ByRun/sra/SRR/SRR106/SRR1067560
HUES64 cell line	SRR1067578	ftp://ftp.ncbi.nlm.nih.gov/sra/sra-instant/reads/ByRun/sra/SRR/SRR106/SRR1067578
IMR90 cell line	SRR019650	ftp://ftp.ncbi.nlm.nih.gov/sra/sra-instant/reads/ByRun/sra/SRR/SRR019/SRR019650
IMR90 cell line	SRR020121	ftp://ftp.ncbi.nlm.nih.gov/sra/sra-instant/reads/ByRun/sra/SRR/SRR020/SRR020121
IMR90 cell line	SRR020102	ftp://ftp.ncbi.nlm.nih.gov/sra/sra-instant/reads/ByRun/sra/SRR/SRR020/SRR020102
iPS DF 19.11 cell line	SRR179609	ftp://ftp.ncbi.nlm.nih.gov/sra/sra-instant/reads/ByRun/sra/SRR/SRR179/SRR179609
iPS DF 19.11 cell line	SRR179610	ftp://ftp.ncbi.nlm.nih.gov/sra/sra-instant/reads/ByRun/sra/SRR/SRR179/SRR179610
iPS DF 19.11 cell line	SRR179611	ftp://ftp.ncbi.nlm.nih.gov/sra/sra-instant/reads/ByRun/sra/SRR/SRR179/SRR179611
iPS DF 6.9 cell line	SRR179595	ftp://ftp.ncbi.nlm.nih.gov/sra/sra-instant/reads/ByRun/sra/SRR/SRR179/SRR179595
iPS DF 6.9 cell line	SRR179596	ftp://ftp.ncbi.nlm.nih.gov/sra/sra-instant/reads/ByRun/sra/SRR/SRR179/SRR179596
iPS DF 6.9 cell line	SRR179615	ftp://ftp.ncbi.nlm.nih.gov/sra/sra-instant/reads/ByRun/sra/SRR/SRR179/SRR179615
large intestine	SRR770593	ftp://ftp.ncbi.nlm.nih.gov/sra/sra-instant/reads/ByRun/sra/SRR/SRR770/SRR770593
large intestine	SRR770594	ftp://ftp.ncbi.nlm.nih.gov/sra/sra-instant/reads/ByRun/sra/SRR/SRR770/SRR770594
large intestine	SRR770595	ftp://ftp.ncbi.nlm.nih.gov/sra/sra-instant/reads/ByRun/sra/SRR/SRR770/SRR770595
liver	SRR641603	ftp://ftp.ncbi.nlm.nih.gov/sra/sra-instant/reads/ByRun/sra/SRR/SRR641/SRR641603
liver	SRR641604	ftp://ftp.ncbi.nlm.nih.gov/sra/sra-instant/reads/ByRun/sra/SRR/SRR641/SRR641604
liver	SRR641605	ftp://ftp.ncbi.nlm.nih.gov/sra/sra-instant/reads/ByRun/sra/SRR/SRR641/SRR641605
lung	SRR536237	ftp://ftp.ncbi.nlm.nih.gov/sra/sra-instant/reads/ByRun/sra/SRR/SRR536/SRR536237
lung	SRR536238	ftp://ftp.ncbi.nlm.nih.gov/sra/sra-instant/reads/ByRun/sra/SRR/SRR536/SRR536238
lung	SRR1045636	ftp://ftp.ncbi.nlm.nih.gov/sra/sra-instant/reads/ByRun/sra/SRR/SRR104/SRR1045636
muscle leg	SRR1067579	ftp://ftp.ncbi.nlm.nih.gov/sra/sra-instant/reads/ByRun/sra/SRR/SRR106/SRR1067579
muscle leg	SRR1067581	ftp://ftp.ncbi.nlm.nih.gov/sra/sra-instant/reads/ByRun/sra/SRR/SRR106/SRR1067581

muscle leg	SRR1067584	ftp://ftp.ncbi.nlm.nih.gov/sra/sra-instant/reads/ByRun/sra/SRR/SRR106/SRR1067584
muscle trunk	SRR1104843	ftp://ftp.ncbi.nlm.nih.gov/sra/sra-instant/reads/ByRun/sra/SRR/SRR110/SRR1104843
muscle trunk	SRR1104844	ftp://ftp.ncbi.nlm.nih.gov/sra/sra-instant/reads/ByRun/sra/SRR/SRR110/SRR1104844
muscle trunk	SRR1104849	ftp://ftp.ncbi.nlm.nih.gov/sra/sra-instant/reads/ByRun/sra/SRR/SRR110/SRR1104849
Neurosphere Cultured Cells Cortex Derived	GSM1127118	ftp://ftp.ncbi.nlm.nih.gov/geo/samples/GSM1127nnn/GSM1127118/suppl/GSM1127118%5FUCSF%2DUBC%2ENeurosphere%5FCultured%5FCells%5FCortex%5FDerived%2EBisulfite%2DSeq%2EHuFNSC02%2Ewig%2Egz
Neurosphere Cultured Cells Cortex Derived	GSM1127124	ftp://ftp.ncbi.nlm.nih.gov/geo/samples/GSM1127nnn/GSM1127124/suppl/GSM1127124%5FUCSF%2DUBC%2ENeurosphere%5FCultured%5FCells%5FCortex%5FDerived%2EBisulfite%2DSeq%2EHuFNSC04%2Ewig%2Egz
Neurosphere Cultured Cells Ganglionic Eminence Derived	GSM1127055	ftp://ftp.ncbi.nlm.nih.gov/geo/samples/GSM1127nnn/GSM1127055/suppl/GSM1127055%5FUCSF%2DUBC%2ENeurosphere%5FCultured%5FCells%5FGanglionic%5FEminence%5FDerived%2EBisulfite%2DSeq%2EHuFNSC02%2EA17784%2Ewig%2Egz
Neurosphere Cultured Cells Ganglionic Eminence Derived	GSM1127121	ftp://ftp.ncbi.nlm.nih.gov/geo/samples/GSM1127nnn/GSM1127121/suppl/GSM1127121%5FUCSF%2DUBC%2ENeurosphere%5FCultured%5FCells%5FGanglionic%5FEminence%5FDerived%2EBisulfite%2DSeq%2EHuFNSC04%2Ewig%2Egz
Neurosphere Cultured Cells Ganglionic Eminence Derived	GSM941746	ftp://ftp.ncbi.nlm.nih.gov/geo/samples/GSM941nnn/GSM941746/suppl/GSM941746%5FUCSF%2DUBC%2ENeurosphere%5FCultured%5FCells%5FGanglionic%5FEminence%5FDerived%2EBisulfite%2DSeq%2EHuFNSC02%2EA13819%2Ewig%2Egz
ovary	SRR577609	ftp://ftp.ncbi.nlm.nih.gov/sra/sra-instant/reads/ByRun/sra/SRR/SRR577/SRR577609
ovary	SRR577610	ftp://ftp.ncbi.nlm.nih.gov/sra/sra-instant/reads/ByRun/sra/SRR/SRR577/SRR577610
ovary	SRR1045705	ftp://ftp.ncbi.nlm.nih.gov/sra/sra-instant/reads/ByRun/sra/SRR/SRR104/SRR1045705
pancreas	SRR536245	ftp://ftp.ncbi.nlm.nih.gov/sra/sra-instant/reads/ByRun/sra/SRR/SRR536/SRR536245
pancreas	SRR536246	ftp://ftp.ncbi.nlm.nih.gov/sra/sra-instant/reads/ByRun/sra/SRR/SRR536/SRR536246
pancreas	SRR1045706	ftp://ftp.ncbi.nlm.nih.gov/sra/sra-instant/reads/ByRun/sra/SRR/SRR104/SRR1045706
Penis Foreskin Fibroblast Primary Cells	GSM1127120	ftp://ftp.ncbi.nlm.nih.gov/geo/samples/GSM1127nnn/GSM1127120/suppl/GSM1127120%5FUCSF%2DUBC%2EPenis%5FForeskin%5FFibroblast%5FPrimary%5FCells%2EBisulfite%2DSeq%2Eskin03%2Ewig%2Egz

Penis Foreskin Keratinocyte Primary Cells	GSM1127056	ftp://ftp.ncbi.nlm.nih.gov/geo/samples/GSM1127nnn/GSM1127056/suppl/GSM1127056%5FUCSF%2DUBC%2EPenis%5FForeskin%5FKeratinocyte%5FPrimary%5FCells%2EBisulfite%2DSeq%2Eskin03%2Ewig%2Egz
Penis Foreskin Keratinocyte Primary Cells	GSM1127058	ftp://ftp.ncbi.nlm.nih.gov/geo/samples/GSM1127nnn/GSM1127058/suppl/GSM1127058%5FUCSF%2DUBC%2EPenis%5FForeskin%5FKeratinocyte%5FPrimary%5FCells%2EBisulfite%2DSeq%2Eskin03%2Ewig%2Egz
placenta	SRR1104861	ftp://ftp.ncbi.nlm.nih.gov/sra/sra-instant/reads/ByRun/sra/SRR/SRR110/SRR1104861
placenta	SRR1104865	ftp://ftp.ncbi.nlm.nih.gov/sra/sra-instant/reads/ByRun/sra/SRR/SRR110/SRR1104865
placenta	SRR1104866	ftp://ftp.ncbi.nlm.nih.gov/sra/sra-instant/reads/ByRun/sra/SRR/SRR110/SRR1104866
psoas muscle	SRR577625	ftp://ftp.ncbi.nlm.nih.gov/sra/sra-instant/reads/ByRun/sra/SRR/SRR577/SRR577625
psoas muscle	SRR1045645	ftp://ftp.ncbi.nlm.nih.gov/sra/sra-instant/reads/ByRun/sra/SRR/SRR104/SRR1045645
psoas muscle	SRR1045712	ftp://ftp.ncbi.nlm.nih.gov/sra/sra-instant/reads/ByRun/sra/SRR/SRR104/SRR1045712
sigmoid colon	SRR536234	ftp://ftp.ncbi.nlm.nih.gov/sra/sra-instant/reads/ByRun/sra/SRR/SRR536/SRR536234
sigmoid colon	SRR536235	ftp://ftp.ncbi.nlm.nih.gov/sra/sra-instant/reads/ByRun/sra/SRR/SRR536/SRR536235
sigmoid colon	SRR577638	ftp://ftp.ncbi.nlm.nih.gov/sra/sra-instant/reads/ByRun/sra/SRR/SRR577/SRR577638
small intestine	SRR536231	ftp://ftp.ncbi.nlm.nih.gov/sra/sra-instant/reads/ByRun/sra/SRR/SRR536/SRR536231
small intestine	SRR1045659	ftp://ftp.ncbi.nlm.nih.gov/sra/sra-instant/reads/ByRun/sra/SRR/SRR104/SRR1045659
small intestine	SRR1045718	ftp://ftp.ncbi.nlm.nih.gov/sra/sra-instant/reads/ByRun/sra/SRR/SRR104/SRR1045718
spinal cord	SRR1104845	ftp://ftp.ncbi.nlm.nih.gov/sra/sra-instant/reads/ByRun/sra/SRR/SRR110/SRR1104845
spinal cord	SRR1104860	ftp://ftp.ncbi.nlm.nih.gov/sra/sra-instant/reads/ByRun/sra/SRR/SRR110/SRR1104860
spinal cord	SRR1104864	ftp://ftp.ncbi.nlm.nih.gov/sra/sra-instant/reads/ByRun/sra/SRR/SRR110/SRR1104864
spleen	SRR536247	ftp://ftp.ncbi.nlm.nih.gov/sra/sra-instant/reads/ByRun/sra/SRR/SRR536/SRR536247
spleen	SRR1045660	ftp://ftp.ncbi.nlm.nih.gov/sra/sra-instant/reads/ByRun/sra/SRR/SRR104/SRR1045660
spleen	SRR1045724	ftp://ftp.ncbi.nlm.nih.gov/sra/sra-instant/reads/ByRun/sra/SRR/SRR104/SRR1045724
stomach	SRR1104840	ftp://ftp.ncbi.nlm.nih.gov/sra/sra-instant/reads/ByRun/sra/SRR/SRR110/SRR1104840
stomach	SRR1104846	ftp://ftp.ncbi.nlm.nih.gov/sra/sra-instant/reads/ByRun/sra/SRR/SRR110/SRR1104846

stomach	SRR1104847	ftp://ftp.ncbi.nlm.nih.gov/sra/sra-instant/reads/ByRun/sra/SRR/SRR110/SRR1104847
thymus	SRR577606	ftp://ftp.ncbi.nlm.nih.gov/sra/sra-instant/reads/ByRun/sra/SRR/SRR577/SRR577606
thymus	SRR1045667	ftp://ftp.ncbi.nlm.nih.gov/sra/sra-instant/reads/ByRun/sra/SRR/SRR104/SRR1045667
thymus	SRR1067580	ftp://ftp.ncbi.nlm.nih.gov/sra/sra-instant/reads/ByRun/sra/SRR/SRR106/SRR1067580
UCSF-4 embryonic stem cell line	GSM1127122	ftp://ftp.ncbi.nlm.nih.gov/geo/samples/GSM1127nnn/GSM1127122/suppl/GSM1127122%5FUCSF%2DUBC%2EUCSF%2D4star%2EBisulfite%2DSeq%2EA21771%2Ewig%2Egz
UCSF-4 embryonic stem cell line	GSM1127123	ftp://ftp.ncbi.nlm.nih.gov/geo/samples/GSM1127nnn/GSM1127123/suppl/GSM1127123%5FUCSF%2DUBC%2EUCSF%2D4star%2EBisulfite%2DSeq%2EA21772%2Ewig%2Egz
